# *LncSox4* promotes the self-renewal of liver tumour-initiating cells through Stat3-mediated Sox4 expression

**DOI:** 10.1038/ncomms12598

**Published:** 2016-08-24

**Authors:** Zhen-zhen Chen, Lan Huang, Ya-hong Wu, Wen-jie Zhai, Ping-ping Zhu, Yan-feng Gao

**Affiliations:** 1 School of Life Sciences, Zhengzhou University, Zhengzhou 450001, China; 2 The First Affiliated Hospital, Zhengzhou University, Zhengzhou 450052, China; 3 School of Life Sciences, University of Science and Technology of China, Hefei, Anhui 230027, China; 4 Collaborative Innovation Center of New Drug Research and Safety Evaluation, Zhengzhou 450001, Henan Province, China

## Abstract

Liver cancer has a tendency to develop asymptomatically in patients, so most patients are diagnosed at a later stage. Accumulating evidence implicates that liver tumour-initiating cells (TICs) as being responsible for liver cancer initiation and recurrence. However, the molecular mechanism of liver TIC self-renewal is poorly understood. Here we discover that a long noncoding RNA (lncRNA) termed *LncSox4* is highly expressed in hepatocellular carcinoma (HCC) tissues and in liver TICs. We find that *LncSox4* is required for liver TIC self-renewal and tumour initiation. *LncSox4* interacts with and recruits Stat3 to the *Sox4* promoter to initiate the expression of Sox4, which is highly expressed in liver TICs and required for liver TIC self-renewal. The expression level of Sox4 correlates with HCC development, clinical severity and prognosis of patients. Altogether, we find that *LncSox4* is highly expressed in liver TICs and is required for their self-renewal.

## Abstract (web summary)

Liver tumour-initiating cells (TICs) may be responsible for liver cancer initiation and recurrence. In this article, the authors show that a previously unidentified lncRNA, LncSox4, is highly expressed in liver cancer TICs and regulates TIC self-renewal through the Stat3/SOX4 axis.

## Graphical abstract

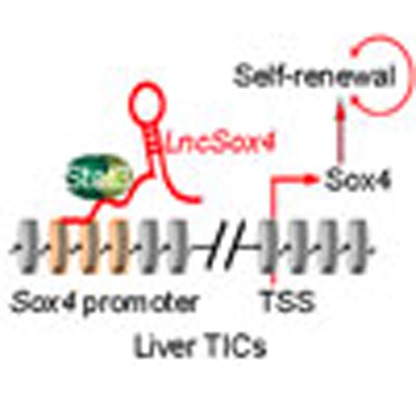

Hepatocellular carcinoma (HCC) is the most common type of liver cancer, the third leading cause of tumour-related death^1^. Like other tumours, HCC tissues contain a heterogeneous mixture of cells^2^. Tumour-initiating cells (TICs), a small subset within tumour bulk, are considered to be the source of tumours^3^. Liver TICs are responsible for liver cancer formation, metastasis, drug-resistance and recurrence^4^. Recently, several liver TIC markers have been identified, including EPCAM, CD133, CD13, CD90, CD24 and calcium channel α2δ1 subunit^5^,^6^. In addition, several signalling pathways are known to be involved in liver TIC self-renewal, including Notch, Wnt/β-catenin, Hedgehog and TGFβ signalling pathways^7^,^8^,^9^,^10^. Given the critical role of TICs in tumorigenesis, intervention of TIC self-renewal can be a potential strategy for tumour treatment^11^. However, the biological characters and molecular mechanisms of liver TICs formation and maintenance are still poorly understood.

Long noncoding RNAs (lncRNAs) are defined as a type of RNA molecules that are longer than 200 nucleotides (nt) without protein coding potential. Due to the lack of corresponding proteins, lncRNAs were once considered as byproducts of RNA polymerase II, without biological functions. Recently the critical roles of lncRNAs in physiological and pathological process have been frequently discovered. LncRNAs are involved in cell and organ development, stemness maintenance, cell differentiation and X chromatin inactivation^12^,^13^,^14^. In addition, lncRNAs also participate in tumour proliferation, metastasis, invasion, energy regulation and TIC self-renewal^7^,^8^,^15^,^16^,^17^. Mechanistically, lncRNAs exert their roles through *cis*-regulation or *trans*-regulation^18^. LncRNAs can interact with chromatin DNA, mRNA or protein, thus regulating chromatin accessibility, mRNA stability and protein activity or stability^18^,^19^. Though more and more lncRNAs have been discovered and critical roles of lncRNAs emerge, the biological function and molecular mechanism of lncRNAs are largely unknown.

Transcription factors, especially several stem factors have been reported in liver TIC regulation, including Oct4, Nanog, c-Myc and Zic2 (refs 20, 21, 22). Stat3, a well-known transcription factor, plays critical roles in development, inflammation, stemness regulation and tumorigenesis^14^,^23^,^24^,^25^. Sox4, a Sox family transcription factor, is thought to be required for nervous system development, epigenetic reprogramming, stemness regulation and tumorigenesis^26^,^27^,^28^. However, the function of Stat3 and Sox4 in liver tumorigenesis and liver TIC self-renewal is largely unknown.

Here we discovered a long noncoding RNA termed *LncSox4* initiates liver TIC self-renewal through Stat3–Sox4 pathway. *LncSox4* interacts with and recruits Stat3 to *Sox4* promoter, initiating Sox4 expression and liver TIC self-renewal. We proved that the Stat3–Sox4 pathway participates in liver tumorigenesis and liver TIC self-renewal, thus offering a new potential target of TICs for eradicating liver cancer.

## Results

### *LncSox4* is highly expressed in liver cancer and liver TICs

**Figure 1:**
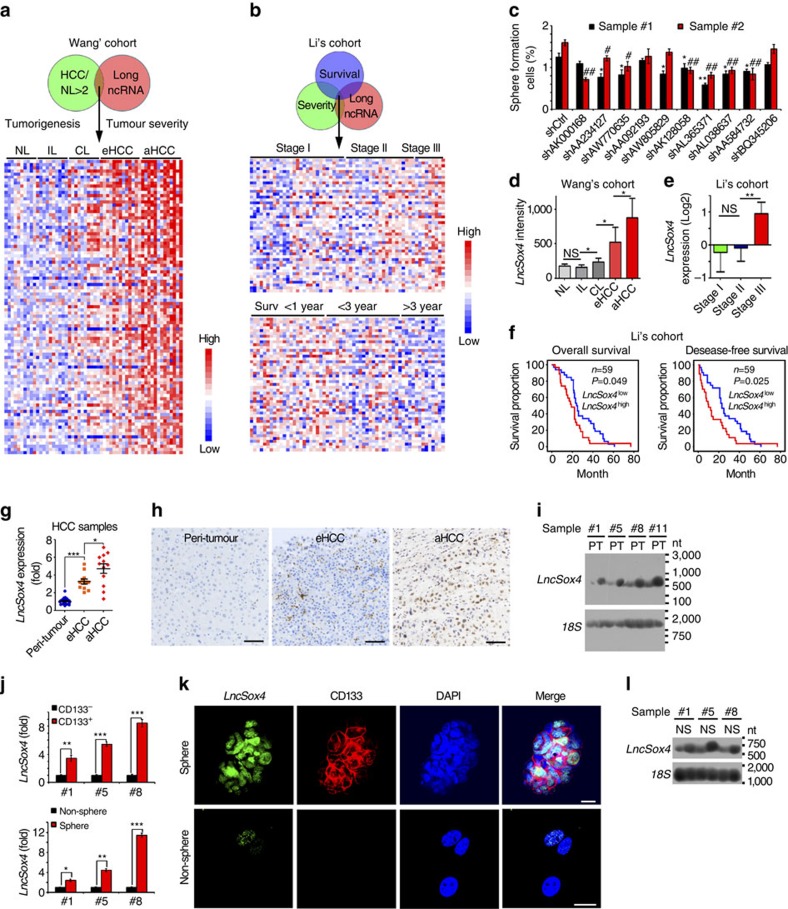
*LncSox4* is highly expressed in liver cancer and liver TICs. (**a**) Expression profiles of lncRNAs in HCC samples were analysed using R language and Bioconductor according to Wang's cohort. NL, normal liver; IL, inflammatory liver; CL, cirrhosis liver; eHCC, early HCC; aHCC, advanced HCC. (**b**) Li's cohort was used to analyse lncRNAs that are related to clinical severity and prognosis. (**c**) *LncSox4* plays an essential role in liver TIC self-renewal. 10 lncRNAs that are highly expressed in liver cancer and related to HCC severity and prognosis were selected. (**d**) High expression of *LncSox4* in liver cancer. *LncSox4* expression was analysed with R language and Bioconductor, and expression levels of *LncSox4* were shown as mean±s.d. (**e**,**f**) *LncSox4* expression levels were related to clinical stages (**e**) and prognosis (**f**) according to Li's cohort. Samples were divided into different groups according to clinical severity (**e**) or *LncSox4* expression level (**f**), followed by expression analysis (**e**) or Kaplan–Meier survival analyses (**f**). (**g**–**i**) High expression of *LncSox4* in liver cancer was confirmed by primary HCC samples. The indicated HCC samples were used for *LncSox4* expression analysis using real-time PCR (**g**), *in situ* hybridization (**h**) and northern blot (**i**). (**j**) Histogram (mean±s.d.) of *LncSox4* in liver TICs and non-TICs. CD133^+^ liver TICs were sorted (upper panel) and oncospheres were derived (lower panel) from the indicated primary samples, followed by real-time PCR assays for *LncSox4* transcript #1, primary sample #1. (**k**,**l**) High expression of *LncSox4* was confirmed by FISH (**k**) and northern blot (**l**). FITC-conjugated *LncSox4* probes and anti-CD133 antibody were used for non-sphere and sphere staining, followed by counterstain with DAPI. N, Non-sphere; S, Sphere. Scale bars, H, 100 μm; K, 10 μm. Data are shown as means±s.d. Two-tailed Student's *t*-test was used for statistical analysis. *P<0.05; **P<0.01; ***P<0.001; ^#^*P*<0.05; ^##^*P*<0.01; NS, not significant. *P*<0.05 was considered significant. Data are representative of at least three independent experiments. For **i** and **l**, the original images are shown in Supplementary Fig. 6.

To understand how liver TICs are formed and maintained during tumorigenesis, we analysed several online-available data sets using R language and Bioconductor approaches^29^. Surprisingly, we found that many uncharacterized lncRNAs correlated to tumorigenesis and clinical outcomes of HCC patients. For example, in Wang's cohort (GSE54238, microarray data of lncRNAs and genes, containing 10 normal liver samples, 10 inflammatory liver samples, 10 cirrhosis liver samples, 13 early HCC samples and 13 advanced HCC samples)^30^, hundreds of lncRNAs are highly expressed in HCC tissues and related to liver tumorigenesis. Top 100 lncRNAs were selected and a heatmap was generated (Fig. 1a). We also analysed another patient cohort, Li's cohort (GSE40144, microarray data of genes and lncRNAs, with disease-free survival and overall survival information of 59 hepatocellular carcinoma), and found hundreds of lncRNAs related to HCC severity and prognosis. We chose top 50 lncRNAs highly expressed in advanced samples and top 50 lncRNAs highly expressed in HCC patients with poor prognosis (Fig. 1b). On the basis of the overlap of these selections, we finally focused on 10 lncRNAs that are highly expressed in liver cancer and also related to both HCC severity and prognosis. To examine the effects of these lncRNAs on TIC self-renewal, we established lncRNA-silenced cells using pSiCoR lentivirus, followed by sphere formation, a standard approach to detect TIC self-renewal. We found the weakest sphere formation capacity of AL365371 (hereafter termed *LncSox4*) depleted cells, indicating the critical role of *LncSox4* in liver TIC self-renewal (Fig. 1c). Furthermore, *LncSox4* is highly expressed in HCC tissues (Fig. 1d), especially advanced HCC patients (Fig. 1e). Importantly, patients with higher *LncSox4* expression levels had poorer outcomes, and vice versa (Fig. 1f). We then examined the expression levels of *LncSox4* using 24 peri-tumour, 12 early HCC and 12 advanced HCC samples by real-time PCR (Fig. 1g). Furthermore, *in situ* hybridization (ISH) assays were performed using 30 peri-tumour, 12 early HCC and 18 advanced HCC samples (Fig. 1h and Supplementary Fig. 1A), confirming high *LncSox4* expression in liver tumours, especially in advanced liver tumours. We also observed similar results using northern blot (Fig. 1i). Above all, *LncSox4* is highly expressed in liver tumours and related to clinical severity and prognosis.

Considering the critical role of *LncSox4* in liver TIC self-renewal, we next examined *LncSox4* expression levels in liver TICs. First, we analysed the expression profiles of *LncSox4* and liver TIC surface markers. The samples were divided into two groups according to the expression levels of two widely accepted liver TIC surface markers, *EPCAM* and *CD133* (mean expression levels of *EPCAM* and *CD133* served as cut-off values, respectively), and then *LncSox4* expression levels were analysed. Higher expression levels of *LncSox4* were found in *EPCAM*^high^ and *CD133*^high^ samples, suggesting that *LncSox4* is highly expressed in liver TICs (Supplementary Fig. 1B). The positive correlation between *LncSox4* and TIC markers was confirmed in primary HCC samples, indicating that *LncSox4* is related to liver TICs (Supplementary Fig. 1C). CD133 was used to enrich liver TICs and non-TICs. CD133^+^ cells showed vigorous sphere formation and tumour initiation capacities, conferring CD133^+^ cells as liver TICs (Supplementary Fig. 1D,E and Supplementary Table 1A). Then we enriched liver TICs from primary HCC samples by flow cytometer, detected *LncSox4* expression levels in CD133^-^ (non-TICs) and CD133^+^ (TICs) cells, and found higher expression of *LncSox4* in liver TICs (Fig. 1j, upper panels). To determine *LncSox4* expression in stem-like oncospheres and non-spheres derived from primary samples, we performed sphere formation assays using primary HCC cells and measured the expression levels of *LncSox4* by real-time PCR, confirming the high expression of *LncSox4* in stem-like oncospheres (Fig. 1j, lower panels). We verified the expression profiles of *LncSox4* using fluorescence *in situ* hybridization (FISH) and confirmed high expression levels of *LncSox4* in oncospheres (Fig. 1k). Finally we analysed *LncSox4* expression in liver TICs using northern blot. Three samples were used for sphere formation and followed by northern blot, validating the high expression levels of *LncSox4* in liver TICs (Fig. 1l). Another lncRNA (*BC016831*) showed comparable expression levels in liver TICs compared with non-TICs, and spheres compared with non-spheres, indicating specific *LncSox4* expression in liver TICs (Supplementary Fig. 1F). To determine the location of *LncSox4* in liver TICs, we performed nucleocytoplasmic separation using stem-like oncospheres, and examined the content of *LncSox4* in nuclear and cytoplasmic fractions using real-time PCR (Supplementary Fig. 1G). The results showed that *LncSox4* is located in the cell nucleus, consistent with the ISH results in Fig. 1h and FISH results in Fig. 1k. Altogether, *LncSox4* is highly expressed in HCC and liver TICs.

### *LncSox4* is essential for liver TIC self-renewal

**Figure 2:**
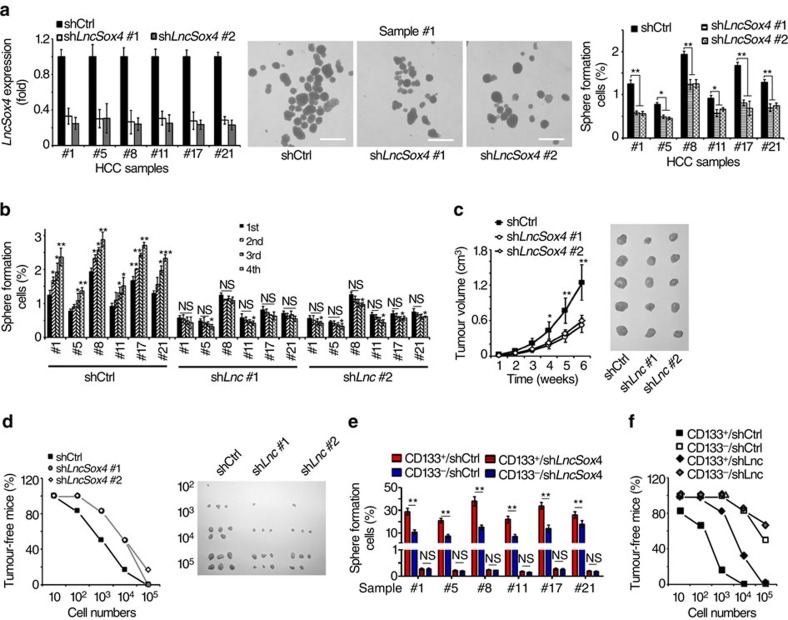
*LncSox4* is required for liver TIC self-renewal. (**a**) *LncSox4* plays an essential role in sphere formation. *LncSox4*-silenced primary HCC cells were established (left panel) using pSiCoR lentivirus, followed by sphere formation assays. Representative sphere pictures were shown in middle panels and calculated TIC ratios were shown in the right panels. Scale bars, 500 μm. (**b**) Serial sphere formation of *LncSox4*-silenced (shLnc) and control (shCtrl) primary HCC cells. For each sphere formation assays, 5,000 primary HCC cells (the 1st generation sphere formation) or signal sphere cells (the second, third, fourth generations) were used. (**c**) *LncSox4* is required for HCC propagation. A total of 1 × 10^6^ *LncSox4*-silenced or control cells were subcutaneously injected into BALB/c nude mice on the back, and tumour volumes were measured at the indicated time points. The tumour pictures were shown in right panels. (**d**) *LncSox4* participates in HCC initiation. 10, 1 × 10^2^, 1 × 10^3^, 1 × 10^4^ and 1 × 10^5^ *LncSox4*-silenced or control cells were subcutaneously injected into BALB/c nude mice on the back and tumour formation was observed three months later. Tumour-free mice ratios and tumour pictures were shown. (**e**,**f**) sphere formation (**e**) and tumour-initiation (**f**) assays of *LncSox4*-silenced liver TICs. CD133^+^ liver TICs and CD133^−^ non-TICs were enriched from primary HCC cells using flow cytometer, followed by infection with *LncSox4-*silenced and control pSiCoR lentivirus. For sphere formation, 5,000 cells were incubated in FBS-free medium for 2 weeks. For tumour initiation, 10, 1 × 10^2^, 1 × 10^3^, 1 × 10^4^ and 1 × 10^5^ cells were subcutaneously injected into BALB/c nude mice on the back for 3 months. Data are shown as means±s.d. Two tailed Student's *t*-test was used for statistical analysis. *P<0.05; **P<0.01; ***P<0.001; NS, not significant. *P*<0.05 was considered significant. Data are representative of three independent experiments.

Considering high *LncSox4* expression in liver TICs, we further analysed its role in liver TIC self-renewal. First, we established *LncSox4*-silenced primary HCC cells using pSiCoR lentivirus, and the knockdown efficiency was confirmed using real-time PCR (Fig. 2a, left panels). Spheres with diameter >100 μm were counted 2 weeks later. *LncSox4*-depleted cells showed impaired sphere formation capacity, indicating the critical role of *LncSox4* in liver TIC self-renewal (Fig. 2a, middle and right panels). Notably, there was no obvious difference of sphere size between *LncSox4-*silenced and control spheres (Supplementary Fig. 2A). We further detected the role of *LncSox4* in liver TIC self-renewal using serial sphere formation assays. Four generations of sphere formation were performed, showing impaired long-term self-renewal with *LncSox4* knockdown (Fig. 2b). To further examine the role of *LncSox4* in liver TIC self-renewal, we silenced *LncSox4* in 30 HCC samples available, and found impaired self-renewal in 23 samples using sphere formation assays (Supplementary Fig. 2B). What is more, we co-stained the spheres with antibodies against OCT4, a widely used stemness transcription factor, and Ki67, a well-known proliferation marker, showing impaired stemness and proliferation in *LncSox4*-silenced spheres (Supplementary Fig. 2C).

Then 1 × 10^6^ *LncSox4*-silenced cells and control cells were subcutaneously injected into BALB/c nude mice, and tumour volumes were measured at the indicated time points. The tumour growth curves and tumour pictures indicate that *LncSox4* is required for *in vivo* propagation of liver cancer (Fig. 2c). To detect tumour initiation, 10, 1 × 10^2^, 1 × 10^3^, 1 × 10^4^ and 1 × 10^5^ cells were subcutaneously injected into BALB/c nude mice and tumour initiation was evaluated 3 months later. Tumour-free mice ratios and established tumours were shown in Fig. 2d. TIC ratios were calculated using extreme limiting dilution analysis. The results showed that *LncSox4*-silenced cells contain remarkably low ratios of liver TICs, suggesting the essential role of *LncSox4* in tumour initiation (Supplementary Table 1B). To further examine the role of *LncSox4* in liver TICs, liver TICs and non-TICs were enriched and infected with *LncSox4-*silencing short hairpin RNA (shRNA) and control shRNA lentivirus. Sphere formation assays showed impaired self-renewal of *LncSox4-*silenced TICs, while, in non-TICs, *LncSox4* knockdown had impaired effect on sphere formation, indicating specific role of *LncSox4* in liver TICs (Fig. 2e). Similarly, tumour initiation assays also confirmed the critical role of *LncSox4* in liver TICs (Fig. 2f, Supplementary Table 1C).

We further verified the role of *LncSox4* in liver TICs using *LncSox4* overexpression cells (Supplementary Fig. 2D, upper panels). *LncSox4* overexpression enhanced sphere formation (Supplementary Fig. 2D, middle and lower panels), but had no obvious impact on sphere size (Supplementary Fig. 2E), confirming the essential role of *LncSox4* in liver TICs. We also detected long-term self-renewal of liver TICs using serial sphere formation, and found enhanced sphere formation capacities of *LncSox4* overexpressing cells, confirming the critical role of *LncSox4* in liver TIC self-renewal (Supplementary Fig. 2F). Then 1 × 10^6^ *LncSox4* overexpressed and control cells were subcutaneously injected into BALB/c nude mice, further confirming the critical role of *LncSox4* in tumour propagation and initiation (Supplementary Fig. 2G). In summary, *LncSox4* is essential for liver TIC self-renewal, tumour initiation and propagation.

### *LncSox4* promotes liver TIC self-renewal through Sox4

**Figure 3:**
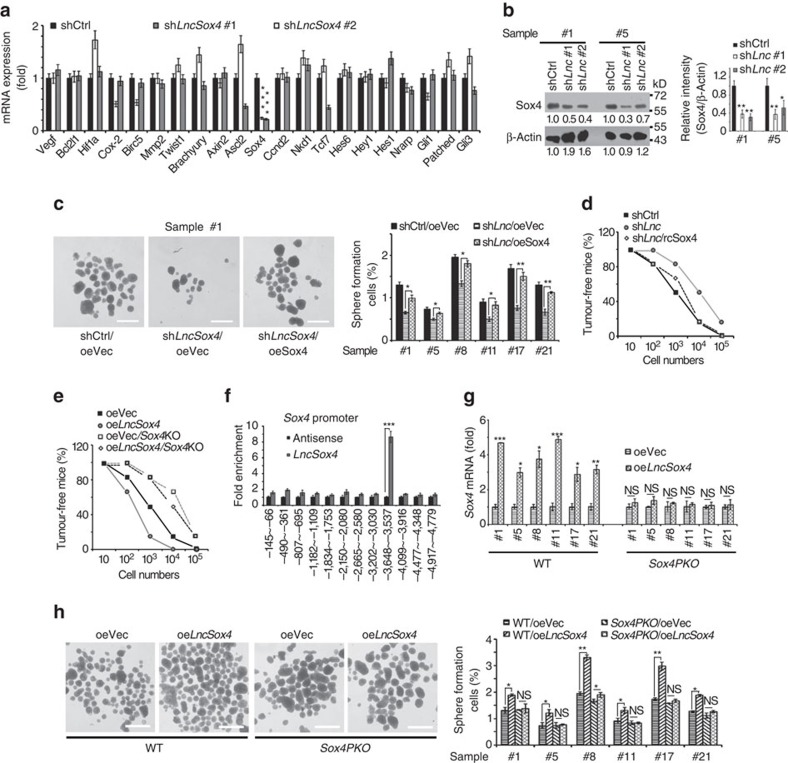
*LncSox4* drives liver TIC self-renewal through Sox4. (**a**,**b**) *LncSox4* is required for Sox4 expression. mRNA expression of the indicated genes in *LncSox4*-silenced (sh*LncSox4*) and control cells (shCtrl) were examined using real-time PCR (**a**). Impaired Sox4 expression in *LncSox4*-silenced cells was confirmed by western blot (**b**). (**c**,**d**) *LncSox4* drives liver TIC self-renewal through Sox4 expression. Sox4 expression was rescued using PBPLV lentivirus in *LncSox4*-silenced cells, and then sphere formation (**c**) and tumour initiation (**d**) assays were performed. Five thousand indicated primary HCC cells were used for sphere formation, and 10, 1 × 10^2^, 1 × 10^3^, 1 × 10^4^ and 1 × 10^5^ cells were subcutaneously injected into BALB/c nude mice for tumour initiation. (**e**) Primary HCC cells were treated with CRISPR/Cas9 lentivirus for *Sox4* deficiency, followed by *LncSox4* overexpression (oeLnc). The indicated cells were used for *in vivo* tumour initiation, and the ratios of tumour-free mice were calculated 3 months later. (**f**) *LncSox4* binds to *Sox4* promoter. RNA ChIP assay was performed and fold enrichment was examined using real-time PCR. (**g**,**h**) The interaction of *LncSox4* and *Sox4* promoter was required for *LncSox4* function. The *Sox4* promoter region for *LncSox4* binding (−3,647∼−3,537) was deleted using CRISPR/Cas9 approach (*Sox4PKO*), followed by *LncSox4* overexpression. The established cells were examined for *Sox4* expression and sphere formation capacities. Six samples were examined and similar results were found. Scale bars, C, H 500 μm. Data were shown as means±s.d. Two-tailed Student's *t*-test was used for statistical analysis. *P<0.05; **P<0.01; ***P<0.001. *P*<0.05 was considered significant. Data are representative of three independent experiments. For **b**, the original images are shown in Supplementary Fig. 6. NS, not significant.

To explore the molecular mechanism of *LncSox4* in liver TICs, we established *LncSox4*-silenced cells to identify its target genes. It is widely accepted that NFκB, Wnt/β-catenin, Notch and Hedgehog signalling pathways play critical roles in TIC self-renewal. Therefore, we checked the role of *LncSox4* in the activation of these signalling pathways. We examined the expression levels of NFκB target genes (Vegf, Bcl2l1, Hif1a, Birc5, Mmp2, Twist1), Wnt/β-catenin target genes (Myc, Tiam, Kiaa, Ccnd1, Ccnd2, Sox4, Fn14, Tcf1), Notch target genes (Hes6, Hey1, Hes1, Nrarp) and Hedgehog target genes (Gli1, Patched, Gli3); and found impaired expression of transcription factor Sox4 on *LncSox4* knockdown (Fig. 3a). To rule out off-target effects of shRNA treatment, we designed two independent *LncSox4* shRNAs to confirm the target genes. The genes (such as *Tcf7*) downregulated by only one *LncSox4* shRNA are probably off-target genes. The impaired Sox4 expression was further confirmed by western blot (Fig. 3b). We then analysed the expression correlation between *LncSox4* and *Sox4* using online-available data sets. The samples were divided into two groups according to *LncSox4* or *Sox4* expression levels (mean expression levels served as cut-off values), and we found high Sox4 expression in *LncSox4*^high^ samples, and vice versa (Supplementary Fig. 3A,B). The positive correlation between *LncSox4* and *Sox4* expression was also confirmed using primary HCC samples (Supplementary Fig. 3C). These data indicated that *LncSox4* could drive *Sox4* expression.

Next, we examined whether *LncSox4* promotes liver TIC self-renewal through Sox4. We silenced Sox4 expression and found reduced self-renewal by sphere formation assays, indicating the critical role of Sox4 in liver TIC self-renewal (Supplementary Fig. 3D). Then we rescued Sox4 expression in *LncSox4*-silenced cells and examined sphere formation. Sox4 rescue remarkably neutralized the impaired sphere formation induced by *LncSox4* depletion, indicating *LncSox4* drives liver TIC self-renewal in a Sox4-dependent manner (Fig. 3c). What is more, 10, 1 × 10^2^, 1 × 10^3^, 1 × 10^4^ and 1 × 10^5^ *LncSox4*-silenced and Sox4 rescued cells were subcutaneously injected into BALB/c nude mice and tumour initiation was examined 3 months later. We found Sox4 largely rescued the impaired tumour initiation capacities induced by *LncSox4* knockdown (Fig. 3d, Supplementary Table 1D). In addition, we established *Sox4* knockout cells using CRIPSR/Cas9 approach and overexpressed *LncSox4* in *Sox4* knockout cells, followed by tumour initiation assays using gradient transplant model. In wild-type (WT) cells, *LncSox4* overexpression significantly promoted tumour initiation, while *LncSox4* overexpression failed to promote tumour formation in *Sox4* knockout cells, validating the critical role of Sox4 in *LncSox4-*mediated liver TIC activation (Fig. 3e, Supplementary Table 1E).

To examine whether *LncSox4* regulates *Sox4* expression through combination with *Sox4* promoter, ChIRP assay was performed and enrichment of *Sox4* promoter regions was measured using real-time PCR. The result suggested the interaction between *LncSox4* and *Sox4* promoter (Fig. 3f). We also examined *Cox-2*, *Birc5*, *Ascl2* and *Tcf7* promoters, but did not find an obvious enrichment, confirming the specific binding between *LncSox4* and Sox4 promoter. Meanwhile, the failure of *LncSox4-*promoter binding conferred these genes as ‘off targets' of *LncSox4* shRNAs (Supplementary Fig. 3E). In addition, we predicted the structure of *LncSox4* using thermodynamic ensemble and minimum free energy methods. Loops usually play critical roles in lncRNA function, so we focused on loops and found several common loops using two prediction methods. Surprisingly, the nucleotide sequence of one loop is highly complementary to the *LncSox4* binding region of *Sox4* promoter (Supplementary Fig. 3F), indicating the combination of *LncSox4* and *Sox4* promoter through sequence complement. We then deleted the *LncSox4* binding region of *Sox4* promoter using CRISPR/Cas9 approach (Supplementary Fig. 3G,H), and examined the effect of *LncSox4* on Sox4 expression and liver TIC self-renewal. *LncSox4* positively regulates Sox4 expression and liver TIC self-renewal in WT cells. Interestingly, *LncSox4* overexpression had no longer impacted on *Sox4* expression (Fig. 3g) and sphere formation (Fig. 3h) in *LncSox4* binding region-deleted cells, suggesting that the interaction between *LncSox4* and *Sox4* promoter is essential for *LncSox4*-triggered Sox4 expression and liver TIC self-renewal.

Here, we discovered that *LncSox4* binds to *Sox4* promoter via sequence complement, which plays an essential role in *LncSox4* function. LncRNA can bind to gene promoter to regulate gene expression in *cis*, but the molecular mechanism of this regulation mode is rarely reported. By CRISPR/Cas9-mediated editing of *Sox4* promoter, we proved that *LncSox4* binding to *Sox4* promoter through sequence complement. Using Sox4 overexpression/knockout systems and sphere formation assays, we also proved that this complementary sequence is required for *LncSox4* function.

### *LncSox4* interacts with Stat3

**Figure 4:**
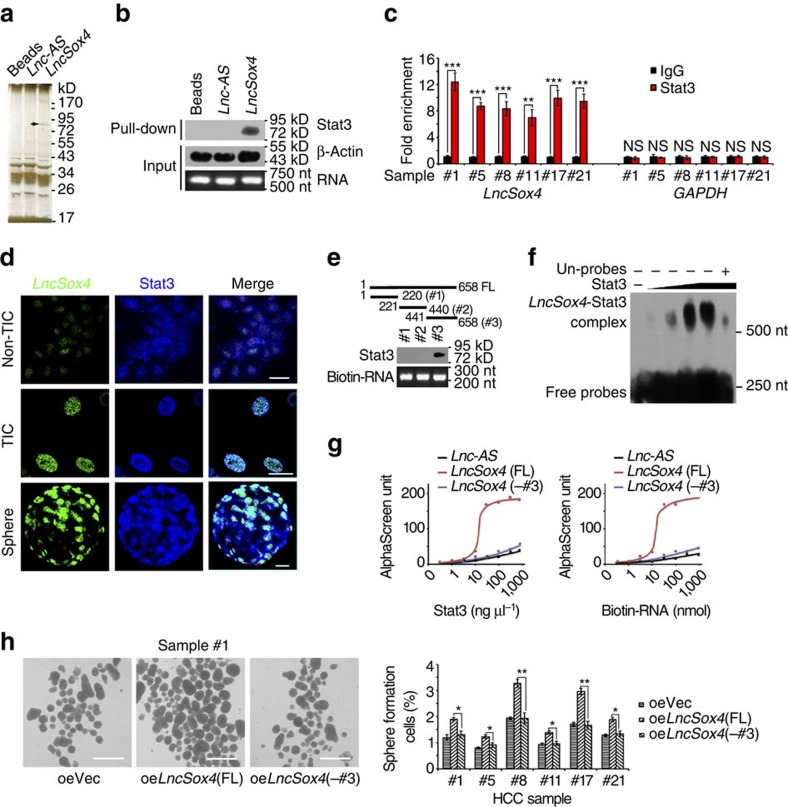
*LncSox4* interacts with Stat3. (**a**) RNA pull-down assay was performed using *LncSox4*, *Lnc-AS* (*LncSox4* antisense RNA) and control RNA, and the samples were separated by Coomassie staining, followed by identification using mass spectrum. The black arrow indicates Stat3. KD, kiloDalton. (**b**) The interaction between *LncSox4* and Stat3 was confirmed by RNA pull-down and western blot. Lnc-AS, *LncSox4* anti-sense. β-Actin served as loading controls. (**c**) RIP was performed using anti-Stat3 and control IgG antibodies, followed by real-time PCR to examine the enrichment of *LncSox4* and GAPDH. GAPDH served as a negative control. (**d**) *LncSox4* is co-localized with Stat3. *LncSox4* probes and anti-Stat3 antibody were used to examine the subcellular location using flow cytometer sorted CD133^-^ (Non-TIC), CD133^+^ (TIC) cells and stem-like oncospheres (Sphere). Scale bars, 10 μm. (**e**) Three regions of *LncSox4* were examined for interaction with Stat3. The indicated truncates of *LncSox4* were constructed and RNA pull-down assays were performed. The samples were examined by western blot with anti-Stat3 antibody. (**f**) *LncSox4* interacts with Stat3. Biotin-labelled *LncSox4* was obtained by *in vitro* transcription assays and incubated with recombination Stat3 protein, followed by RNA EMSA. 100 × unlabelled probes were used for competitive EMSA. (**g**) Bead-based proximity assay of *LncSox4*, *LncSox4* truncate (-#3 region) and *LncSox4*-antisense *(Lnc-AS*) (50 nnol) were incubated with various concentrations (horizontal axis) of Stat3 (left) or Stat3 (50 ng μl^−1^) mixed with various concentrations (horizontal axis) of biotin-labelled RNA (right); and units generated by the assay system were shown. (**h**) *LncSox4* (FL), *LncSox4* truncate (-#3 region) overexpressing primary HCC cells were established, followed by sphere-formation assay. Typical spheres were shown in left panels, and sphere formation ratios in right panels. Scale bars, 500 μm. Data were shown as means±s.d. Two-tailed Student's *t*-test was used for statistical analysis. *P<0.05; **P<0.01; ***P<0.001. *P*<0.05 was considered significant. Data are representative of four independent experiments. For **b**, **e** and **f**, the original images are shown in Supplementary Fig. 6. NS, not significant.

To further identify the molecular mechanism and binding partners of *LncSox4* in liver TICs, we performed RNA pull-down assays followed by silver staining. *LncSox4* specific bands were identified by mass spectrum, and Stat3 were found to interact with *LncSox4* (Fig. 4a). The samples were further analysed using western blot, confirming the interaction of *LncSox4* and Stat3 (Fig. 4b). We also verified this result using RNA immunoprecipitation (RIP) and found *LncSox4* could be enriched in Stat3 precipitates, further proving that *LncSox4* interacts with Stat3 (Fig. 4c). The interaction between *LncSox4* and Stat3 was also confirmed by double FISH. *LncSox4* is highly expressed in TICs and oncospheres and located within the cell nucleus. Notably, obvious co-localization of *LncSox4* and Stat3 was found in liver TICs and spheres (Fig. 4d and Supplementary Fig. 4A). In addition, the interaction region of *LncSox4* toward Stat3 was identified using *LncSox4* truncates, revealing the essential role of region #3 in the combination between *LncSox4* and Stat3 (Fig. 4e). RNA electrical mobility shift assay (RNA EMSA) was performed using biotin-labelled probes (corresponding to region #3), validating the interaction of *LncSox4* and Stat3 (Fig. 4f). We also performed amplified luminescent-proximity homogeneous assay to validate their interaction. The interactions of RNA and gradient Stat3 or Stat3 and gradient RNA were analysed, showing the relatively strong interaction between Stat3 and *LncSox4*, but not *LncSox4* truncate or *LncSox4-*antisense (Fig. 4g). This result further confirms the specific binding of *LncSox4* and Stat3, and the critical role of region #3 in their binding.

Finally, we examined the biological relevance of the interaction between *LncSox4* and Stat3. We detected the protein levels of Stat3 and found comparable protein levels in *LncSox4*-silenced and control cells, indicating that *LncSox4* has no effect on Stat3 stability (Supplementary Fig. 4B). To examine the role of *LncSox4*–Stat3 association in *LncSox4* function, we overexpressed *LncSox4* and *LncSox4* truncate without Stat3 binding region, and found impaired function of *LncSox4* truncate in sphere formation (Fig. 4h). Altogether, *LncSox4* associates with Stat3.

### *LncSox4* is required for Stat3-induced Sox4 expression

**Figure 5:**
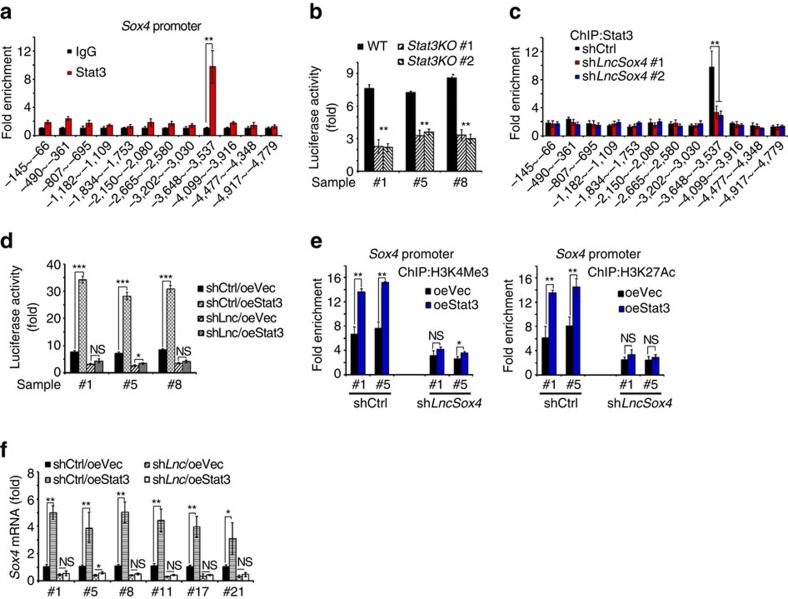
*LncSox4* is required for Stat3 binding to *Sox4* promoter. (**a**) Stat3 binds to *Sox4* promoter. ChIP assays were performed using anti-Stat3 and control IgG antibodies and fold enrichment of the indicated regions of *Sox4* promoter was examined using real-time PCR. (**b**) Stat3 is required for *Sox4* promoter activation. *Stat3*-deficient cells were established using CRISPR/Cas9 system. *Sox4* promoter was constructed into PGL3 luciferase reporter vector and transfected into *Stat3*-deficient and control cells. Luciferase triggered by *Sox4* promoter activation was measured. (**c**) *LncSox4* is required for the interaction between Stat3 and *Sox4* promoter. Stat3 ChIP assays were performed using *LncSox4*-depleted cells, and enrichment of the indicated regions of *Sox4* promoter was examined using real-time PCR. (**d**) *LncSox4* is required for Stat3-induced activation of *Sox4* promoter. Stat3 were overexpressed in *LncSox4*-impaired cells, and then luciferase was examined. Enhanced activation of *Sox4* promoter was found upon Stat3 overexpression in control cells, while, comparable activation levels were observed in *LncSox4*-silenced cells. (**e**) Stat3 were overexpressed in *LncSox4*-silenced cells, followed by ChIP with antibodies against H3K4Me3 (left panels) and H3K27Ac (right panels), and fold enrichment of *Sox4* promoter was examined using real-time PCR. oe, overexpression. (**f**) *LncSox4* is required for Stat3-induced *Sox4* expression. Stat3 were overexpressed in *LncSox4*-depleted cells, and then *Sox4* expression levels were detected using real-time PCR. Stat3 overexpression induced higher *Sox4* expression in control cells but comparable *Sox4* expression in *LncSox4*-silenced cells. Data were shown as means±s.d. Two-tailed Student's *t*-test was used for statistical analysis. *P<0.05; **P<0.01; ***P<0.001. *P*<0.05 was considered significant. Data are representative of four independent experiments. NS, not significant.

We then investigated the role of Stat3 in *LncSox4*-induced Sox4 expression. First, we detected whether Stat3 binds to *Sox4* promoter using ChIP assay, and found that a specific region of the *Sox4* promoter was enriched upon Stat3 immunoprecipitation, indicating that Stat3 binds to *Sox4* promoter and probably regulate Sox4 expression (Fig. 5a). To further characterize the role of Stat3 in Sox4 expression, we deleted Stat3 using CRISPR/Cas9 approaches and found impaired activation of *Sox4* promoter upon Stat3 deficiency, confirming that Stat3 regulates Sox4 expression (Fig. 5b). Interestingly, Stat3 bound the same region of *Sox4* promoter as *LncSox4* (Figs 3f and 5a), implying the possible function of *LncSox4* in the interaction between Stat3 and *Sox4* promoter. We then performed ChIP assay with Stat3 antibody using *LncSox4*-silenced cells and found remarkably decreased enrichment of *Sox4* promoter upon *LncSox4* depletion, proving the essential role of *LncSox4* in Stat3 binding to *Sox4* promoter (Fig. 5c).

To further verify the function of *LncSox4* in Stat3-mediated Sox4 expression, we overexpressed Stat3 in *LncSox4*-silenced cells, and examined the activity of *Sox4* promoter, chromatin accessibility and Sox4 expression. The Stat3/*LncSox4* binding region of *Sox4* promoter was cloned into PGL3 luciferase reporter plasmid. We found that Stat3 overexpression enhanced the luciferase activity in WT cells, However, Stat3 overexpression could not enhance the *Sox4* promoter activity in *LncSox4* knockdown cells (Fig. 5d). We also performed ChIP assays with antibodies against H3K4me3 and H3K27ac, two histone modification markers widely used for promoter activation examination. We found enhanced activation of *Sox4* promoter on Stat3 overexpression in WT cells but not in *LncSox4*-silenced cells (Fig. 5e). Similarly, Stat3 overexpression significantly increased *Sox4* expression in WT cells but not in *LncSox4*-deficient cells (Fig. 5f). In summary, Stat3 has little effect on *Sox4* promoter activity, transcription activation of *Sox4* promoter and *Sox4* mRNA expression in *LncSox4*-depleted cells, strongly proving that *LncSox4* is essential for Stat3-induced *Sox4* promoter activation and *Sox4* mRNA expression.

### Stat3 and Sox4 are required for liver TIC self-renewal

**Figure 6:**
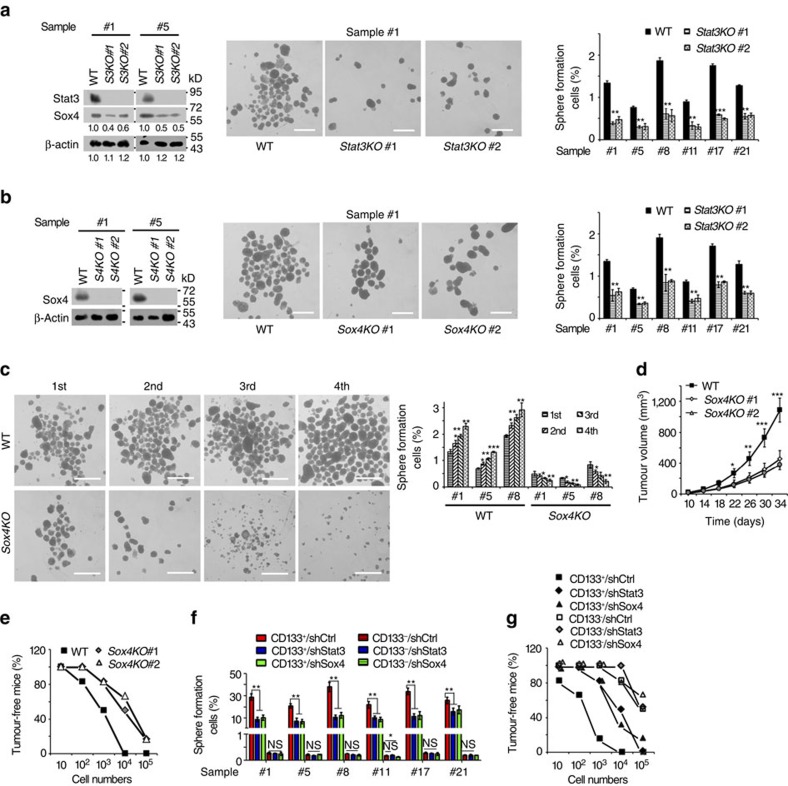
Stat3/Sox4 is required for liver TIC self-renewal. (**a**) Stat3 is required for liver TIC self-renewal. Impaired Sox4 expression (left panels) and sphere formation capacities (middle and right panels) were found in *Stat3*-deficient cells. (**b**) *Sox4*-deficient cells were established and sphere formation assays were performed, and impaired sphere formation was observed. (**c**) Sox4 is essential in liver TIC self-renewal. Serial sphere formation assays were performed using *Sox4*-deficient or control cells. For each sphere formation assay, 5,000 cells were used. Representative images were shown in the left panel and calculated TIC ratios were shown in the right panel. (**d**) Sox4 drives liver cancer propagation. 1 × 10^6^ *Sox4*-deleted primary HCC cells were subcutaneously injected into BALB/c nude mice, and tumour volume was measured at the indicated time points. (**e**) A total 10, 1 × 10^2^, 1 × 10^3^, 1 × 10^4^ and 1 × 10^5^ *Sox4*-deficient primary HCC cells were subcutaneously injected into BALB/c nude mice, and tumour formation was examined 3 months later. (**f**,**g**) Sphere formation (**f**) and tumour initiation (**g**) assays of Stat3- or Sox4-silenced liver TICs. CD133^+^ liver TICs were enriched from primary HCC cells using flow cytometer, followed by infection with Stat3- or Sox4-silenced pSiCoR lentivirus. For **f**, 5,000 cells were incubated in sphere formation medium for 2 weeks. For **g**, 10, 1 × 10^2^, 1 × 10^3^, 1 × 10^4^ and 1 × 10^5^ cells were subcutaneously injected into BALB/c nude mice for tumour-initiation assay. Scale bars, 500 μm (**a**, **b** and **c)**. Data were shown as means±s.d. Two-tailed Student's *t*-test was used for statistical analysis. *P<0.05; **P<0.01; ***P<0.001. *P*<0.05 was considered significant. Data are representative of three independent experiments. For **a** and **b**, the original images are shown in Supplementary Fig. 6. NS, not significant.

Since *LncSox4* primes liver TICs by promoting Stat3-induced Sox4 expression, we then explored the role of Stat3 and Sox4 in liver TIC self-renewal. The relationship between Sox4 and TIC surface markers were analysed using Wang's cohort (GSE14520 (refs 31, 32), with gene microarray data and survival information of two normal liver samples, 228 peri-tumour and 239 liver cancer samples) and Li's cohort (GSE40144). The samples were divided into two groups according to *EPCAM* expression (mean expression level used as cut-off value), and *Sox4* expression levels of these two groups were analysed. High expression levels of *Sox4* were found in *EPCAM*^high^ samples, and vice versa (Supplementary Fig. 5A). CD133, another TIC surface marker, was also analysed and positive correlation between *CD133* and *Sox4* was confirmed as well, indicating high expression of Sox4 in TICs (Supplementary Fig. 5B). We then obtained liver TICs using flow cytometer separation and performed sphere formation assay, followed by *Sox4* mRNA detection. Higher expression levels of *Sox4* were found in CD133^+^ TICs and oncospheres compared with counterparts (Supplementary Fig. 5C). To determine the role of Stat3–Sox4 pathway in TIC self-renewal, we established *Stat3* or *Sox4*-deficient cells using CRISPR/Cas9 approach, and performed sphere formation assays. As expected, impaired Sox4 expression and attenuated sphere formation capacity were found in *Stat3*-deleted cells, indicating the essential role of Stat3 in liver TICs (Fig. 6a). In addition, Stat3 knockout spheres were similar in size to control spheres (Supplementary Fig. 5D). We also silenced Stat3 in 30 primary samples using pSiCoR approaches and found impaired sphere formation capacities in almost all primary HCC samples upon Stat3 depletion (Supplementary Fig. 5E). Finally, we subcutaneously injected 1 × 10^6^ *Stat3* knockout or WT cells into BALB/c nude mice, and observed attenuated tumour expansion upon *Stat3* deficiency (Supplementary Fig. 5F). BALB/c nude mice were subcutaneously injected with the indicated number of *Stat3*-knockout or WT cells to evaluate tumour initiation 3 months later. The impaired tumour initiation capacity was found in mice injected with *Stat3* knockout cells (Supplementary Fig. 5G). More importantly, *LncSox4* overexpression failed to promote liver tumour initiation, indicating *LncSox4* drives liver TIC self-renewal through a Stat3-dependent manner (Supplementary Fig. 5G).

We then compared sphere formation capacity of *Sox4*-deficient and WT cells, and found fewer spheres formed in *Sox4*-deleted cells (Fig. 6b), without obvious changes in the sphere size (Supplementary Fig. 5H), indicating the critical role of Sox4 in sphere formation. In addition, four passages of sphere formation were performed to detect the function of Sox4 in liver TIC self-renewal. WT cells showed enhanced sphere formation capacities in advanced passages, while *Sox4* deficiency impaired sphere formation with fewer spheres obtained in advanced passages. These results demonstrate the critical role of Sox4 in liver TIC self-renewal (Fig. 6c). We also silenced Sox4 in primary HCC cells of 30 samples using pSiCoR lentivirus, and examined sphere-formation capacities. Sox4 knockdown reduced liver TIC self-renewal in 25 HCC samples (Supplementary Fig. 5I). Finally, we detected the role of Sox4 in HCC propagation and initiation. A total 1 × 10^6^ *Sox4*-deficient primary HCC cells were injected into BALB/c nude mice, and tumour volume was measured every 4 days. *Sox4*-deficient cells formed smaller tumours compared with WT cells, indicating the essential role of Sox4 in liver cancer propagation (Fig. 6d). To examine tumour initiation, 10, 1 × 10^2^, 1 × 10^3^, 1 × 10^4^ and 1 × 10^5^ primary HCC cells were subcutaneously injected into BALB/c nude mice and tumour formation was observed 3 months later. We found Sox4-deficient cells initiate fewer tumours compared with the same number of WT cells. Tumour-free mice ratios (Fig. 6e) and TIC ratios (Supplementary Table 1F) proved that Sox4 plays an essential role in tumour initiation.

We then enriched liver TICs by flow cytometer using anti-CD133 antibody, and silenced Stat3 or Sox4 in liver TICs and non-TICs using pSiCoR lentivirus, followed by sphere formation and tumour initiation assays. Stat3 or Sox4 knockdown in TICs largely impaired sphere formation, while Stat3 or Sox4 depletion had little impact on self-renewal in non-TICs (Fig. 6f). In addition, Stat3 or Sox4 depletion in TICs reduced the tumour initiation and TIC ratios, while Stat3 or Sox4 knockdown in non-TICs had little effect on tumour initiation (Fig. 6g, Supplementary Table 1G). It has been reported that Nanog as a Stat3 target gene plays a critical role in TICs, so we compared the role of Nanog and Sox4 in Stat3-mediated liver TIC self-renewal. We rescued Nanog and Sox4 in *Stat3*-deficient cells, and performed sphere-formation assays. Both Nanog and Sox4 served as target genes of Stat3 and played a critical role in Stat3-mediated liver TIC self-renewal (Supplementary Fig. 5J). Above all, the Stat3–Sox4 signalling plays an indispensable role in liver TIC self-renewal, liver cancer initiation and propagation.

### Sox4 is related to HCC severity and prognosis

**Figure 7:**
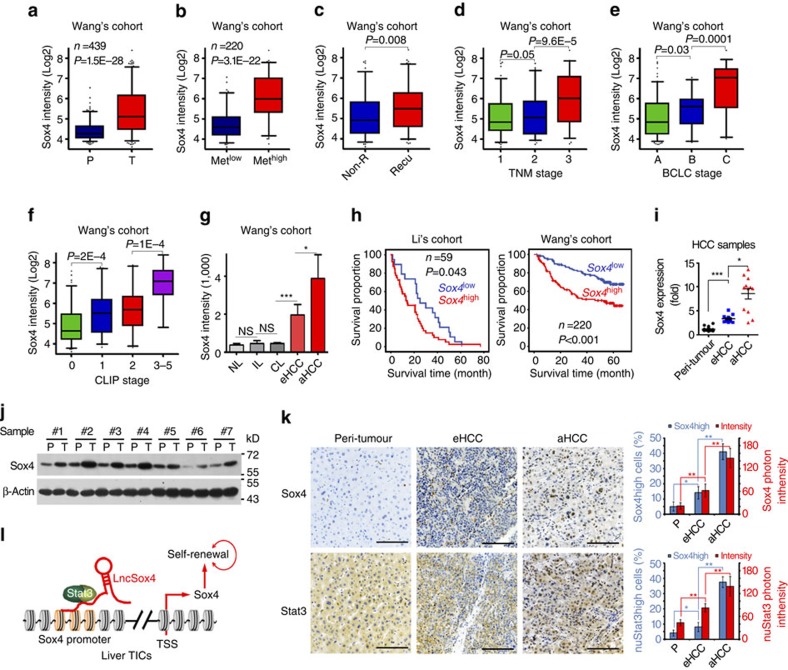
Sox4 expression levels are related to HCC severity and prognosis. (**a**) High expression of *Sox4* in liver cancer tissues according to Wang's cohort. R language and Bioconductor were used for gene expression analysis derived from Wang's cohort. (**b**) *Sox4* is highly expressed in metastasis samples. Tumour samples were divided into two groups according to metastasis properties, and *Sox4* expression levels were analysed. (**c**) *Sox4* expression levels are related to recurrence. Recurrent and non-recurrent samples were selected and *Sox4* expression levels were compared. (**d**–**f**) High expression of *Sox4* in serious HCC samples. The samples were divided according to TNM stage (**d**), BCLC stage (**e**) and CLIP stage (**f**), and *Sox4* expression levels were analysed. BCLC, Barcelona Clinic Liver Cancer; CLIP, Cancer of the Liver Italian Program; TNM, tumour node metastasis. (**g**) Increased *Sox4* expression levels in liver tumorigenesis. aHCC, advanced HCC; CL, cirrhosis liver; eHCC, early HCC; IL, inflammatory liver; NL, normal liver. (**h**) *Sox4* expression is related to HCC prognosis. The samples were grouped according to *Sox4* expression levels and Kaplan–Meier survival analyses were performed. (**i**–**k**) High expression of Sox4 in HCC was confirmed by real-time PCR (**i**), western blot (**j**) and IHC (**k**). Primary HCC samples were obtained and marked according to obtaining time. mRNA was extracted for real-time PCR (**i**), cell lysate was used for western blot (**j**), and formalin-fixed tissue sections were used for IHC (**k**). Scale bars, 100 μm. (**l**) Working model. In liver TICs, *LncSox4* is highly expressed and binds to *Sox4* promoter. *LncSox4* recruits Stat3 to *Sox4* promoter and drives Sox4 expression, which is required for liver TIC self-renewal and related to HCC severity and prognosis. For **a**–**f**, data were shown as box and whisker plot. Box, interquartile range (IQR); whiskers, 5–95 percentiles; horizontal line within box, median. For **g**, **i** and **k**, data were shown as means±s.d. Two-tailed Student's *t*-test was used for statistical analysis. *P<0.05; **P<0.01; ***P<0.001. *P*<0.05 was considered significant. For **j**, the original images are shown in Supplementary Fig. 6. NS, not significant.

Finally we investigated the clinical significance of Sox4 expression in HCC tumorigenesis and progression. We analysed Wang's cohort (GSE14520) using R language and Bioconductor, and found high *Sox4* expression in liver tumours compared with peri-tumour tissues (Fig. 7a). Furthermore, high expression levels of *Sox4* were found in metastasis samples and recurrent samples, indicating the potential role of Sox4 in HCC metastasis and recurrence (Fig. 7b,c). HCC progression was studied using TNM, BCLC and CLIP methods and the samples were divided into different stages on the basis of *Sox4* expression. Higher expression of *Sox4* was found in advanced HCC patients according to TNM stages (Fig. 7d), BCLC stages (Fig. 7e) and CLIP stages (Fig. 7f), indicating that *Sox4* is associated with the severity of HCC. High *Sox4* expression in HCC samples with poor prognosis was also confirmed by another cohort (Fig. 7g). To examine the relationship between *Sox4* expression levels and HCC prognosis, the HCC samples were divided into two groups according to *Sox4* expression levels (mean expression level served as cut-off value), and Kaplan–Meier survival analysis was performed. *Sox4*^high^ patients had a poor prognosis, and *Sox4*^low^ patients could survive longer, proving the critical role of Sox4 in HCC prognosis (Fig. 7h).

We then confirmed these results using primary HCC samples. We detected Sox4 expression levels using real-time PCR (Fig. 7i), western blot (Fig. 7j) and immunohistochemistry (IHC) (Fig. 7k), and confirmed high Sox4 expression in liver cancer, especially in advanced HCC tissues. The cohort data and our own results derived from primary samples endow Sox4 with a critical role in liver tumour. Besides its role in stemness maintenance and development regulation, we uncovered the critical role of Sox4 in liver cancer initiation, progress and prognosis prediction. Taken together, *LncSox4* recruits Stat3 to *Sox4* promoter and drives the expression of Sox4, which is required for liver TIC self-renewal and related to HCC progression, serving as a new target for eradicating liver TICs and a new marker for HCC prognosis (Fig. 7l).

## Discussion

Liver tumour-initiating cells (TICs) account for HCC heterogeneity and recurrence^33^. Many surface markers are used to identify and enrich liver TICs. In this study, we used CD133, a putative surface marker, to isolate liver TICs. CD133 is widely used as a stem marker for neural stem cells, embryonic stem cells, hemopoietic stem cell, glioblastomas and so on^34^,^35^. The shared surface markers between tumour-initiating cells and other stem cells indicate the presence of similar self-renewal mechanism in these stem cells. To determine the self-renewal potential of liver TICs, *in vitro* sphere formation and *in vivo* tumour initiation, two widely used TIC functional assays, were performed in this study. Using these methods, we discovered the essential role of *LncSox4*, Sox4 and Stat3 in liver TICs.

LncRNAs are important for many physiological and pathological processes, including tumorigenesis and stemness^12^,^15^,^36^, so it is reasonable that lncRNAs participate in TIC self-renewal. The functions of lncRNAs are frequently reported, while, their roles in liver TIC self-renewal are largely unknown. To explore specific lncRNAs in liver TIC self-renewal, we analysed online-available data sets with R language and performed ‘unbiased' screening, and discovered many lncRNAs highly expressed in liver tumours and related to clinical prognosis. To determine their roles in liver TIC self-renewal, we performed a shRNA-based function screening and found *LncSox4* related to liver TIC self-renewal. Our results indicate that *LncSox4* promotes liver TIC self-renewal.

Through structure prediction and sequence alignment, we found obvious sequence complement between *LncSox4* loop and *Sox4* promoter. The interaction between *Sox4* promoter and *LncSox4* was confirmed by ChIRP assay. LncRNAs can work through interaction with chromatin DNA, RNA and protein^18^. Many lncRNAs have been reported to interact with proteins and affect their stabilities or activities^14^,^15^,^16^,^17^. Several lncRNAs can bind to gene-promoter regions, while the molecular mechanisms of this binding are rarely reported. Using CRISPR-Cas9 technique, we proved that *LncSox4* binds to *Sox4* promoter through sequence complement. We also proved that *LncSox4* promotes Sox4 expression and liver TIC self-renewal through the sequence complement between *LncSox4* and *Sox4* promoter. Actually, the molecular mechanism of the interaction between *LncSox4* and *Sox4* promoter is still unclear. It is likely that the binding of *LncSox4* and *Sox4* promoter occurs during cell replication when the DNA double strands separate into signal strand. DNA repair and other mechanisms that can trigger the accessibility of double strands are also possible to drive the sequence complement.

Transcription factors are critical for gene transcription and cell fate determination^16^,^37^. Using RNA pull-down assays, we found *LncSox4* interacts with Stat3. Here we found Stat3 binds to *Sox4* promoter and Sox4 is a new target gene of Stat3. Notably, *LncSox4* is required for Stat3 binding to *Sox4* promoter. Stat3 promotes Sox4 expression in WT cells, but not in *LncSox4*-silenced cells. Through these mechanisms, *LncSox4* drives Sox4 expression and liver TIC self-renewal. We also found Stat3–Sox4 signalling pathway is required for liver TIC self-renewal. It has been well documented that Sox4 is crucial in tumorigenesis and stemness, but its function in liver TICs is unclear. We enriched CD133^+^ liver TICs using flow cytometer, and identified that Sox4 is highly expressed in liver TICs. Sox4 deficiency impaired liver TIC self-renewal in sphere formation assays *in vitro* and tumour-initiation experiments *in vivo*. Using online-available cohorts and primary samples, we found Sox4 expression levels related to HCC progression and prognosis, indicating its clinical significance. We also found the transcription factor Stat3 is involved in liver TIC self-renewal. In summary, using sphere formation assays *in vitro* and tumour-initiation assays *in vivo*, we found that the *LncSox4*–Stat3–Sox4 pathway plays a critical role in liver TIC self-renewal and serves as a target for liver TICs eradication.

Altogether, we found *LncSox4* is highly expressed in liver TICs and required for TIC self-renewal. *LncSox4* binds to *Sox4* promoter and recruits Stat3, driving *Sox4* promoter activation and Sox4 expression through *LncSox4*-dependent manner. Sox4 is required for liver TIC self-renewal and can serve as a target for liver TIC eradication. Sox4 expression levels are also related to liver cancer progression and prognosis, providing a new potential marker for HCC diagnosis and prognosis.

## Methods

### Cells and reagents

Human 293T cells were purchased from ATCC and maintained in DMEM medium supplemented with 10% fetal bovine serum (FBS; Invitrogen), 100 μg ml^−1^ penicillin and 100 U ml^−1^ streptomycin. Primary HCC cells were obtained from surgical specimens from the first affiliated hospital of Zhengzhou University with informed consent and institutional approval. Primary samples were numbered according to the time we obtained. Tissues were cut into small pieces and then digested with collagenase IV for 60 min at 37 °C. Primary HCC cells were obtained after centrifugation. All the experiments were approved by the Institutional Committee of Zhengzhou University. HCC clinical information was shown in Supplementary Table 2. The cell lines used in this study were not contaminated by mycoplasma.

Anti-β-Actin (catalogue A1978, 1:1,000) antibody was purchased from Sigma-Aldrich. Anti-Sox4 (catalogue 17919-1-AP, 1:200) antibody was purchased from Proteintech Group. Anti-Oct4 (catalogue 2750, 1:200), anti-Stat3 (catalogue 9139, 1:200), H3K4Me3 (catalogue 9751, 1:500) and H3K27Ac (catalogue 4353, 1:500) antibodies were purchased from Cell Signaling Technology. Phycoerythrin-conjugated CD133 antibody (catalogue 130-098-826, 1:100) was purchased from Miltenyi Biotec. HRP-conjugated secondary antibodies (1:1,000) were purchased from Santa Cruz Biotechnology. Fluorescein-conjugated secondary antibodies were obtained from Molecular Probes Life Technologies.

Other regents used in this study were: DAPI (catalogue 28718-90-3, Sigma-Aldrich), PEG5000 (catalogue 175233-46-2, Sigma-Aldrich), EGF (catalogue E5036-200UG, Life Technologies), N2 supplement (catalogue 17502-048, Life Technologies) and B27 (catalogue 17504-044, Life Technologies), bFGF (catalogue GF446-50UG, Millipore), LightShift Chemiluminescent RNA EMSA Kit (catalogue 20158, Thermo Scientific), Chemiluminescent Nucleic Acid Detection Module (catalogue 89880, Thermo Scientific), T7 RNA polymerase (catalogue 10881767001, Roche), Biotin RNA Labeling Mix (catalogue 11685597910, Roche), ultra low attachment six-well plates (catalogue 3471, Corning), Dual-Luciferase Reporter Assay system (catalogue E1910, Promega).

### Cohort analysis

Online-available data sets were downloaded from EBI ( http://www.ebi.ac.uk/) or NCBI ( http://www.ncbi.nlm.nih.gov/gds/?term=). R language and Bioconductor were used for background correction, normalization, expression calculation and annotation^29^. The gene expression profiles were evaluated for further analyses using Excel, SPSS or Graphpad prism 5 software.

Three online available cohorts were used in this paper. Wang's cohort (GSE54238) shows microarray data of lncRNAs and genes, containing 10 normal liver samples, 10 inflammatory liver samples, 10 cirrhosis liver samples, 13 early HCC samples and 13 advanced HCC samples. Li's cohort (GSE40144) has microarray data of genes and lncRNAs, with disease-free survival and overall survival information of 59 hepatocellular carcinoma. Wang's cohort (GSE14520) has gene microarray data and survival information of two normal liver samples, 228 peri-tumour and 239 liver cancer samples.

### Nucleocytoplasmic separation

A total of 5 × 10^6^ oncosphere cells were resuspended in 0.5 ml resuspension buffer (10 mM HEPES, 1.5 mM MgCl_2_, 10 mM KCl, 0.2% N-octylglucoside, Protease inhibitor cocktail, RNase inhibitor, pH 7.9) for 10 min incubation, followed by homogenization. The cytoplasmic fraction was the supernatant after centrifugation (400*g* × 15 min). The pellet was resuspended in 0.2 ml phosphate-buffered saline (PBS), 0.2 ml nuclear isolation buffer (40 mM Tris-HCl, 20 mM MgCl_2_, 4% Triton X-100, 1.28 M sucrose, pH 7.5) and 0.2 ml RNase-free H_2_O, followed by 20 min incubation on ice to clean out the residual cytoplasmic faction. The pellet was nuclear fraction after centrifugation. RNA was extracted from nuclear and cytoplasmic fractions using RNA extraction kit (Tiangen Company, Beijing). *LncSox4* content was examined by real-time PCR (ABI7300).

For *LncSox4* content, standard reverse transcription was performed using reverse transcription kit (Promega). Notably, same amount of RNA and same volume of cDNA were required. In our experiment, 1 μg nuclear RNA and 1 μg cytoplasmic RNA were used, with the same final volume of nuclear and cytoplasmic cDNA (50 μl). Real-time PCR was performed using 1 μl nuclear cDNA or 1 μl cytoplasmic cDNA, with the same primers and ABI7300 profile. The relative *LncSox4* contents were calculated using these formulae: nuclear ratio=2^−Ct(nuclear)^/(2^−Ct(nuclear)^+2^−Ct(cytoplasmic)^); cytoplasmic ratio=2^−Ct(cytoplasmic)^/(2^−Ct(nuclear)^+2^−Ct(cytoplasmic)^).

### Flow cytometry

Primary HCC cells were incubated with phycoerythrin-conjugated anti-CD133 or control antibodies for 30 min at 4 °C, followed by washing twice in PBS supplemented with 1% FBS. The CD133^+^ and CD133^-^ cells were sorted using FACSArialII cytometer (BD).

### Northern blot

Total RNA was extracted from primary HCC cells or oncospheres according to TRIZOL methods (Invitrogen) for northern blot. LncRNA and 18S fragments were cloned into PCDNA4 plasmid and northern probes were produced using Biotin RNA Labeling Mix (catalogue 11685597910, Roche). T7 RNA polymerase (catalogue 10881767001, Roche) was used for *in vitro* transcription. For northern blot, the samples were separated by electrophoresis using formaldehyde gel, followed by membrane transferring. The membranes were incubated with hydration buffer supplemented with proper amount of probes, and then the nucleic acid signal was detected using Chemiluminescent Nucleic Acid Detection Module (catalogue 89880, Thermo Scientific).

### RNA EMSA assay

Stat3 protein was expressed and purified for RNA EMSA using tandem affinity purification. Biotin-labelled *LncSox4* was produced by *in vitro* transcription using T7 RNA polymerase and Biotin RNA Labeling Mix. Then samples were incubated in the binding buffer according to LightShift Chemiluminescent RNA EMSA Kit (catalogue 20158, Thermo Scientific) for 30 min. The samples were separated by PAGE and Chemiluminescent Nucleic Acid Detection Module was used for signal detection.

### IHC and ISH

HCC and peri-tumour samples were fixed with formalin and embedded with paraffin, and sections were obtained. For IHC and ISH, the samples were incubated in graded alcohols. Then the slides were incubated in 3% hydrogen peroxide (H_2_O_2_) for 30 min. For IHC, the slides were treated with Tris-EDTA buffer (10 mM, pH 8.0) at 121 °C for 5 min for antigen retrieval. The samples were incubated with primary and HRP-conjugated secondary antibodies for IHC, while digoxin-conjugated probes and HRP-conjugated secondary antibodies were used for ISH. Then the samples were co-stained with haematoxylin, followed by dehydration in graded alcohols and xylene. The dilution ratios for IHC were 1:100 for Sox4 antibody, 1:500 for Stat3 antibody, 1:500 for HRP-conjugated secondary antibodies.

### Sphere formation

HCC samples were obtained and digested into single cells with collagenase IV. Five thousand primary HCC cells were seeded into ultra low attachment six-well plates (catalogue 3471, Corning) and incubated in DMEM/F12 medium (Life Technologies) supplemented with 20 ng ml^−1^ EGF, 20 ng ml^−1^ bFGF, N2 supplement and B27. Two weeks later, sphere pictures were taken. Sphere (diameter >100 μm) numbers were counted and sphere formation ratios were calculated using formula (sphere-formation ratio=sphere number/5,000). The spheres and non-spheres were used for further analysis. For serial oncosphere experiment, thespheres were digested into single cells with trypsin/EDTA and 5,000 cells were used for the next generation of sphere formation.

### Real-time PCR

Total RNA was extracted using standard TRIZOL method. The cDNA was obtained using reverse transcription kit (Promega). Expression levels of *LncSox4*, Sox4 and other genes were examined using real-time PCR with ABI7300. 18S and β-Actin served as loading controls. For real-time PCR analyses, we treated all the samples with RNase-free DNase. The primer sequences were shown in Supplementary Table 3.

### Knockdown and knockout

For lncRNA and gene knockdown, siRNA sequence was designed and purchased from Sangon Biotech and then cloned into pSiCoR shRNA vector. For lentivirus production, 293T cells were transfected with pSiCoR, VSVG, pMDL gp RRE and RSV-REV vectors for 3 days. The medium was collected and incubated with PEG5000 for 2 days, and then the virus was collected by centrifugation and used to infect HCC cells. The knockdown cells were established by puromycin selection. The shRNA sequences were shown in Supplementary Table 4.

CRISPR/Cas9 approach was used for *Stat3* and *Sox4* deletion. Guide RNA was designed according to online CRISPR Design Tool ( http://tools. genome-engineering.org) and purchased from Sangon Biotech. Then the sgRNA was cloned into LentiCRISPRv2 (catalogue 52961). For lentivirus production, 293T cells were transfected with LentiCRISPRv2, pVSVG and psPAX2 vectors. The following processes were similar to the knockdown protocol. For *Sox4* promoter deficiency, a pair of guide RNAs was used simultaneously. The infected HCC cells were treated with puromycin and then monoclonalization was performed. The deletion of *Sox4* promoter was confirmed by DNA sequencing.

### FISH analysis

For fluorescence *in situ* hybridization (FISH), fluorescence-conjugated probes were designed by Biosearch Technologies and purchased from Life Technologies. The samples were treated with non-denaturing conditions, followed by adding fluorescence-conjugated probes. For double FISH, primary and secondary antibodies were added. The samples were counterstained with DAPI and observed using confocal microscopy.

### Mouse models

Six-week-old male BALB/c nude mice were purchased from the HFK Biosciences maintained under specific pathogen-free conditions with approval by the Institutional Committee of Zhengzhou University. For tumour propagation, 10^6^ indicated tumour cells were subcutaneously injected into BALB/c nude mice on the back, and then the tumour volume was measured at the indicated time points. For tumour formation assays, 10, 1 × 10^2^, 1 × 10^3^, 1 × 10^4^ and 1 × 10^5^ indicated cells were subcutaneously injected into BALB/c nude mice and the tumour formation was observed 3 months later. TIC ratios were obtained using extreme limiting dilution analysis^38^. Five BALB/c nude mice were used for ervery sample in tumour propagation assays, and six were used for tumour initiation assays.

### ChIP and ChIRP

ChIP assays were performed following standard protocol (Upstate Biotechnology, Inc.). Primary HCC cells or spheres were treated with 1% formaldehyde for 10 min, cracked with SDS lysis buffer followed by ultrasonication, then incubated with proper antibodies (anti-Stat3, anti-H3K4Me3 or anti-H3K27Ac). After washing by high salt, low salt and LiCl buffer, the elution buffer was used to harvest the chromatin fragments. Finally the de-crosslinking was performed and enrichment was examined using real-time PCR.

For ChIRP assays, biotin-labelled *LncSox4* was obtained using *in vitro* transcription and incubated with crashed samples, followed by adding streptavidin-conjugated beads. The following processes were similar to ChIP assays. All buffers used for ChIRP were RNase-free.

### Luciferase assay

The indicated regions of *Sox4* promoter were cloned into pGL3 luciferase reporter plasmid and transfected into the indicated cells. One nanogram pRL-TK was co-transfected as loading control. Thirty-six hours later, the cells were crashed using lysis buffer and detected according to the manual of Dual-Luciferase Reporter Assay system (Promega).

### RNA pull-down and mass spectrometry

Biotin-labelled *LncSox4* was obtained using Biotin RNA Labeling Mix by T7 RNA polymerase, and then incubated with 1% formaldehyde-treated cell lysate for 4 h or overnight. The samples were separated using electrophoresis and *LncSox4* specific bands were identified using mass spectrometry and retrieved in human proteomic library.

### RIP assay

For RIP, the cells were treated with 1% formaldehyde and the crashed with RIPA buffer (150 mM NaCl, 0.5% sodium deoxycholate, 0.1% SDS, 1% NP40, 1 mM EDTA and 50 mM Tris pH 8.0) supplemented with RNase inhibitors and proteinase inhibitors for 30 min, followed by centrifugation. The supernatants were incubated with the indicated antibodies for 4 h, and then protein A/G beads were added. The precipitates were washed with RIPA buffer followed by de-crosslinking. Finally RNA was extracted and *LncSox4* enrichment was examined using real-time PCR.

### Statistics

For box and whisker plots, figures were produced with GraphPad Prism 6 software. Box indicates interquartile range; whiskers indicate 5–95 percentiles; horizontal line within box denotes median value. Two-tailed Student's *t*-test was used for statistical analysis with Microsoft Excel, and *P*<0.05 was considered significant. For histograms, data were shown as means±s.d. Two-tailed Student's *t*-test was used for statistical analysis with Microsoft Excel. *P<0.05; **P<0.01; ***P<0.001. *P*<0.05 was considered significant.

### Data availability

The microarray data referenced during the study (GSE54238, GSE40144 and GSE14520) are available in a public repository from EBI ( http://www.ebi.ac.uk/) or NCBI ( http://www.ncbi.nlm.nih.gov/gds/?term=). All the other data supporting the findings of this study are available within the article and its Supplementary information files. All other relevant source data are available from the corresponding author upon request.

## Additional information

**How to cite this article:** Chen, Z.-z. *et al*. *LncSox4* promotes the self-renewal of liver tumour-initiating cells through Stat3-mediated Sox4 expression. *Nat. Commun.* 7:12598 doi: 10.1038/ncomms12598 (2016).

## Supplementary Material

Supplementary Information Supplementary Figures 1-6 and Supplementary Tables 1-4.
