# Supplementary material for: LncSox4 promotes the self-renewal of liver tumour-initiating cells through Stat3-mediated Sox4 expression
Source: Nat Commun. 2016 Aug 24;7:12598. doi: 10.1038/ncomms12598 (PMC4999516; doi:10.1038/ncomms12598)
Supplement: Supplementary Information — Supplementary Figures 1-6 and Supplementary Tables 1-4. [file ncomms12598-s1.pdf]

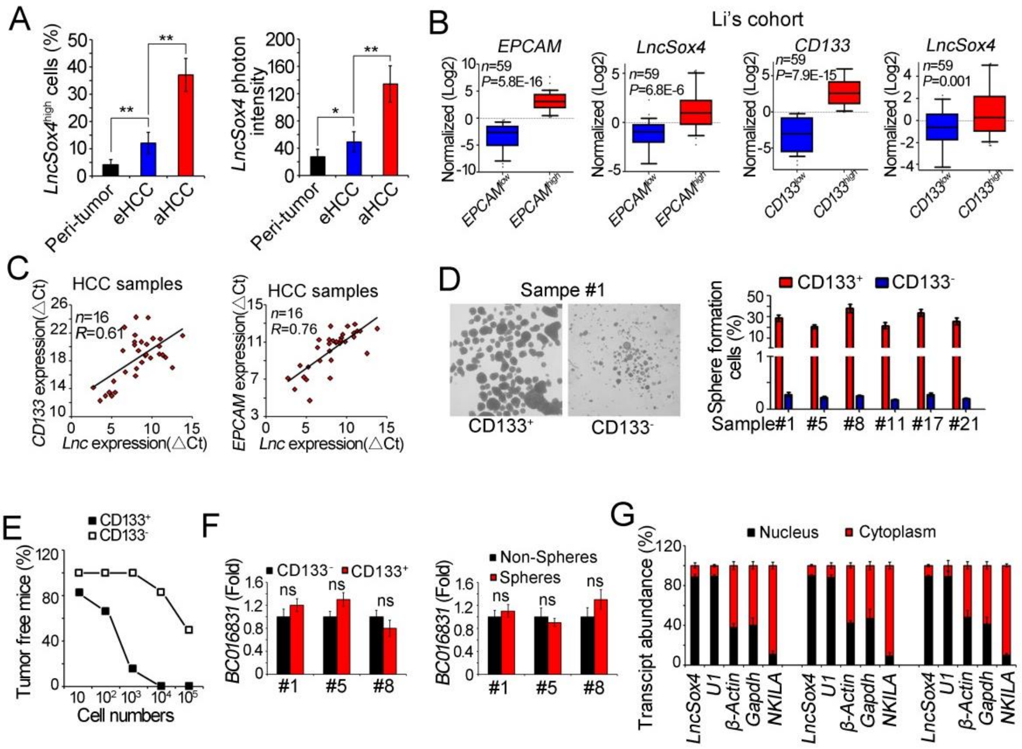

Supplementary Figure 1. *LncSox4* is highly expressed in liver TICs. (A) *LncSox4* ISH results were analyzed with Image-Pro Plus 6, and the ratios of *LncSox4* highly expressed cells and *LncSox4* photon intensity were shown. 30 peri-tumor, 12 early HCC and 18 late HCC samples were used. (B) After analyzing Li's cohort, 59 HCC samples were divided into two groups according to *EPCAM* (left panels) and *CD133* (right panels) expression levels, and then *LncSox4* expression levels were analyzed. Data were shown as box and whisker plot. Box, interquartile range (IQR); whiskers, 5–95 percentiles; horizontal line within box, median. (C) *CD133*, *EPCAM* and *LncSox4* expression levels were examined using real-time PCR and the correction between *LncSox4* and *CD133* (left panel) or *EPCAM* (right panel) were shown. *Lnc, LncSox4*; *R*, Pearson correlation coefficient. (D, E) Sphere formation (D) and tumor initiation (E) by $CD133^+$ and $CD133^-$ cells. HCC primary cells were isolated using collagenase IV, followed by flow cytometer enrichment for $CD133^+$ and $CD133^-$ cells with anti-CD133 antibody. For D, 5000 cells were used for sphere formation. Two weeks later, sphere pictures were taken (left panel) and sphere formation ratios were calculated (right panels). For E, 10, 10^2, 10^3, 10^4 and 10^5 cells were subcutaneously injected into BALB/c nude mice for tumor formation. The ratios of tumor-free mice were shown. CI, confidence interval; vs, versus. P < 0.05 was considered significant. (F) The expression levels of another lncRNA (*BC016831*) in $CD133^+$ liver TICs and oncospheres. Total RNA was extracted from flow cytometer enriched TICs (left panels), or oncospheres (right panels), followed by real-time PCR. (G)

Nucleocytoplasmic separation was performed and the subcellular location of the indicated LncRNAs was measured using real-time PCR. *U1* served as a control of nuclear location, and *NKILA* served as a control of cytoplasm location. For C, D, G, data were shown as means ± SD. Two-tailed Student's t test was used for statistical analysis. $*P < 0.05$; $**P < 0.01$.

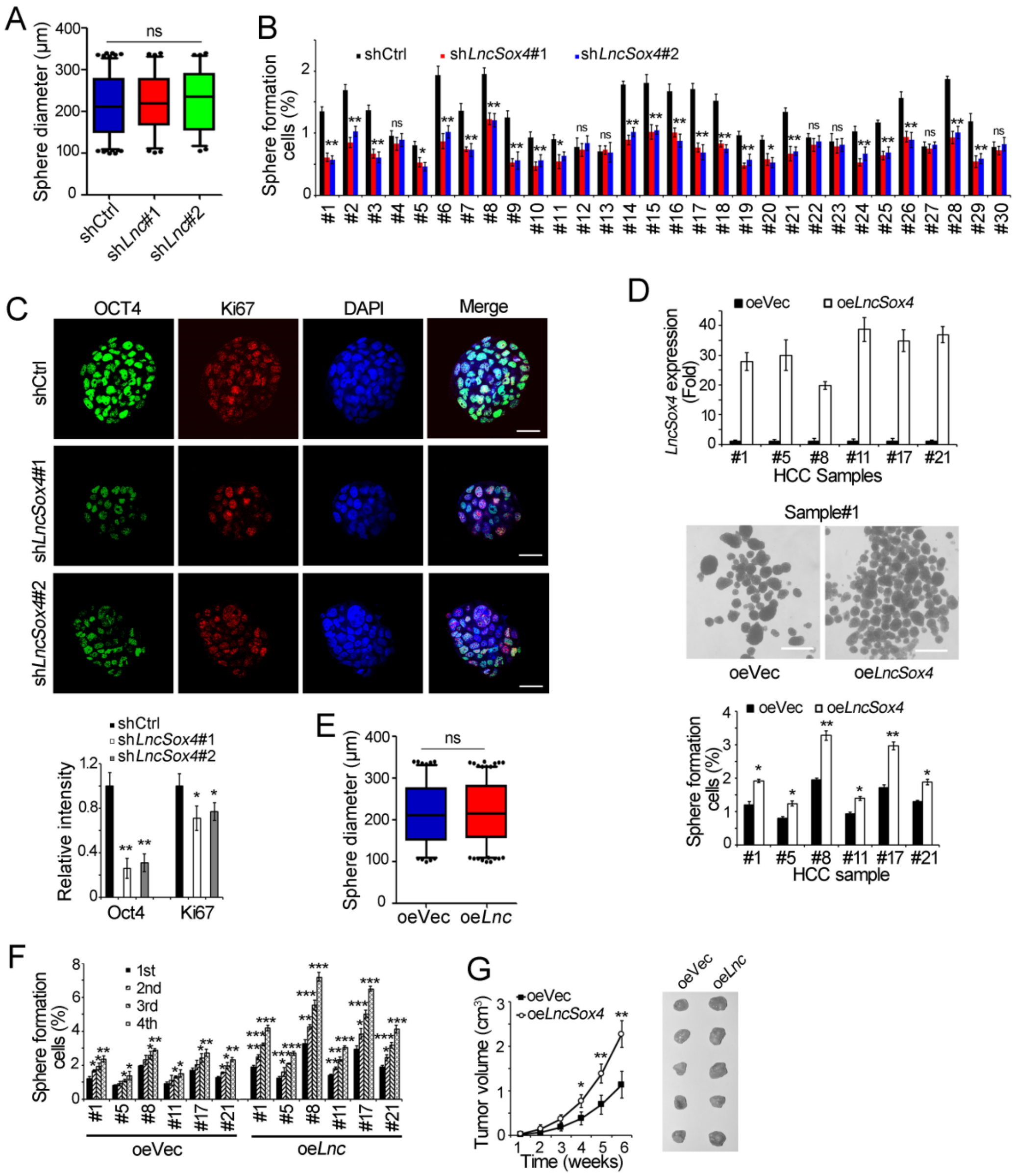

Supplementary Figure 2. *LncSox4* is required for liver TIC self-renewal. (A) The diameter of *LncSox4*-silenced or control spheres was calculated and shown as box and whisker plot. Primary sample #1 cells were used for sphere formation, and the diameter of all spheres (diameter >100 μm) was measured. ns, not significant. (B) Sphere formation ratios of 30 primary samples. 30 primary HCC samples were infected with shSox4 or control pSiCoR lentivirus, followed by flow cytometer sorting for GFP positive cells. 5000 *LncSox4*-silenced or control cells were used for sphere formation. (C) *LncSox4*-silenced or control spheres were used for Oct4 and Ki67 staining, counterstained with DAPI, and observed by confocal microscopy (upper panel). Relative photon intensity of Oct4 (Green) and Ki67 (Red) was calculated and shown as mean ± s.d. Scale bars, 20μm. (D) *LncSox4* promotes liver TIC self-renewal. *LncSox4*-overexpressing primary HCC cells were obtained (upper panel) and used for

sphere formation. Representative images were shown in the middle panel and liver TIC ratios were shown in the lower panel. (E) The diameter of *LncSox4*-overexpressing or control spheres was shown as box and whisker plot. The diameter of all spheres (diameter >100 μm) was measured. ns, not significant. (F) Four generations of sphere formation were performed and sphere formation cells were shown as mean ± SD. For the first generation, 5000 primary HCC cells were used; for the second, third and fourth generations of sphere formation, the former spheres were digested into single cells with Trypsin/EDTA, and 5000 cells were used for the next generation of sphere formation. (G) *LncSox4* promotes liver cancer propagation. 1×10^6 indicated cells were subcutaneously injected into BALB/c nude mice and tumor volumes were measured at the indicated time points. The tumor pictures were shown in the right panel. For A, E, data were shown as box and whisker plot. Box, interquartile range (IQR); whiskers, 5–95 percentiles; horizontal line within box, median. For B, C, D, F, G, data were shown as means ± SD. Two-tailed Student's t test was used for statistical analysis. *$P < 0.05$; **$P < 0.01$, ***$P<0.001$.

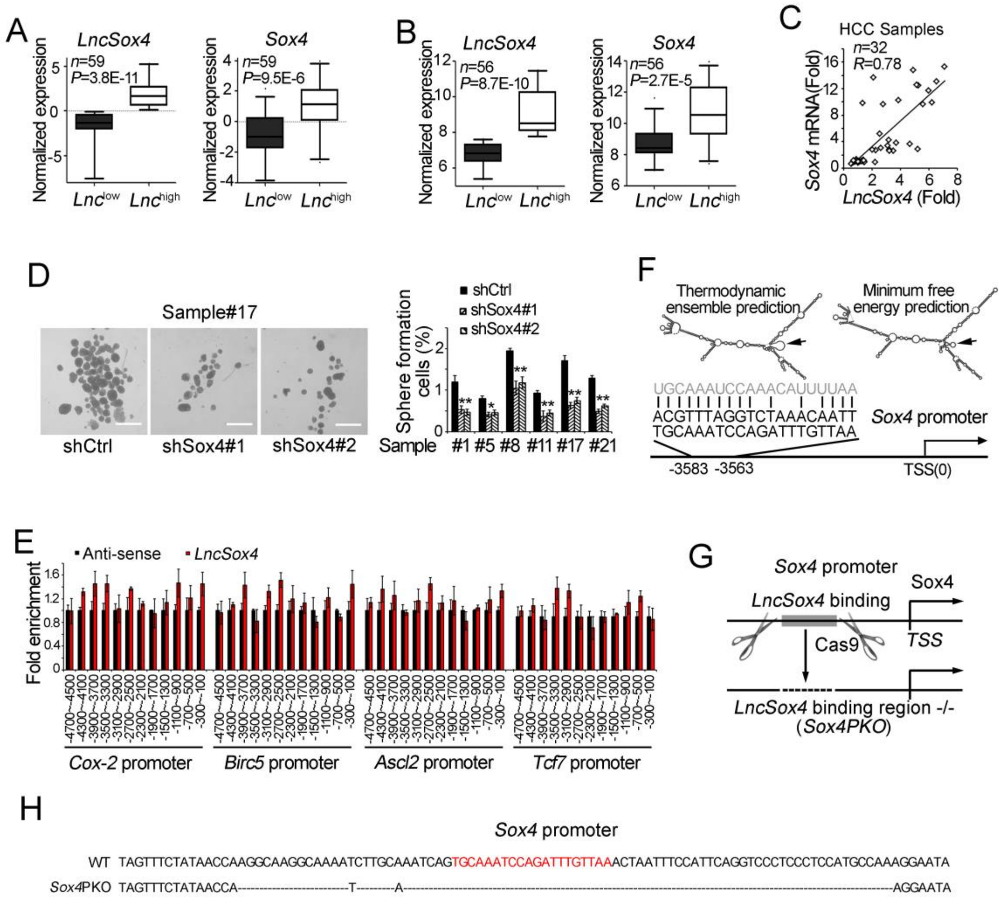

Supplementary Figure 3. *LncSox4* participates in Sox4 expression. (A, B) The relationship between *LncSox4* and Sox4 expression levels was confirmed by Wang's cohort (A) and Li's cohort (B). The samples were divided into two groups according to *LncSox4* expression levels, and Sox4 expression levels of these two groups were analyzed. The cut-off value of Lnc^{low} and Lnc^{high} was the mean value of *LncSox4* expression of all samples. (C) Similar expression profiles of Sox4 and *LncSox4*. The mRNA expression levels of Sox4 and *LncSox4* were examined using real-time PCR and relative fold changes of Sox4 and *LncSox4* expression were shown and liner fit was performed. R, Pearson correlation coefficient. (D) Primary HCC cells were infected with shSox4 or control pSiCoR lentivirus, followed by sphere formation using 5000 indicated cells. Typical images were shown in the left panel and sphere formation ratios were shown as mean ± s.d. Scale bars, 500μm. (E) Fold enrichment of *Cox-2*, *Birc5*, *Ascl2* or *Tcf7* promoter in *LncSox4* or anti-sense control samples. RNA ChIP (ChIRP) were performed using *LncSox4* or anti-sense specific probes. The indicated gene promoter enrichment was analyzed with real-time PCR. (F) The complementary sequence between *LncSox4* and *Sox4* promoter. *LncSox4* structure was predicted according to thermodynamic ensemble prediction and minimum free energy prediction

methods and the nucleotide sequence of the loop domain (black arrow) were shown in gray. The sequence of complementary region of *Sox4* promoter was shown. DNAman was used for sequence alignment. (G, H) *LncSox4* binding region of *Sox4* promoter was deleted using CRISPR/Cas9 method (G), and confirmed by DNA sequencing (H). TSS, transcription start site. Red sequence denotes *LncSox4* binding region. Thereafter *LncSox4* binding region knockout was named Sox4PKO for abbreviation. For A, B, data were shown as box and whisker plot. Box, interquartile range (IQR); whiskers, 5–95 percentiles; horizontal line within box, median. For D, E, data were shown as means ± SD. Two-tailed Student's t test was used for statistical analysis. *P < 0.05; **P < 0.01, ***P<0.001.

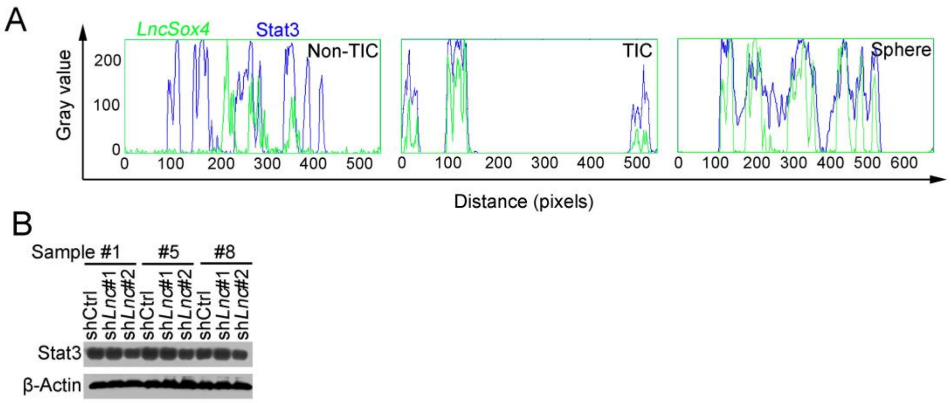

Supplementary Figure 4. *LncSox4* interacts with Stat3. (A) Intensity profiles along the diagonal from upper left to lower right. Green profiles indicate lncSox4 gray value (intensity), and blue profiles indicate Stat3 intensity. (B) *LncSox4* didn't impact Stat3 expressions and stabilities. shCtrl, shControl; shLnc, sh*LncSox4*. β-Actin served as a loading control.

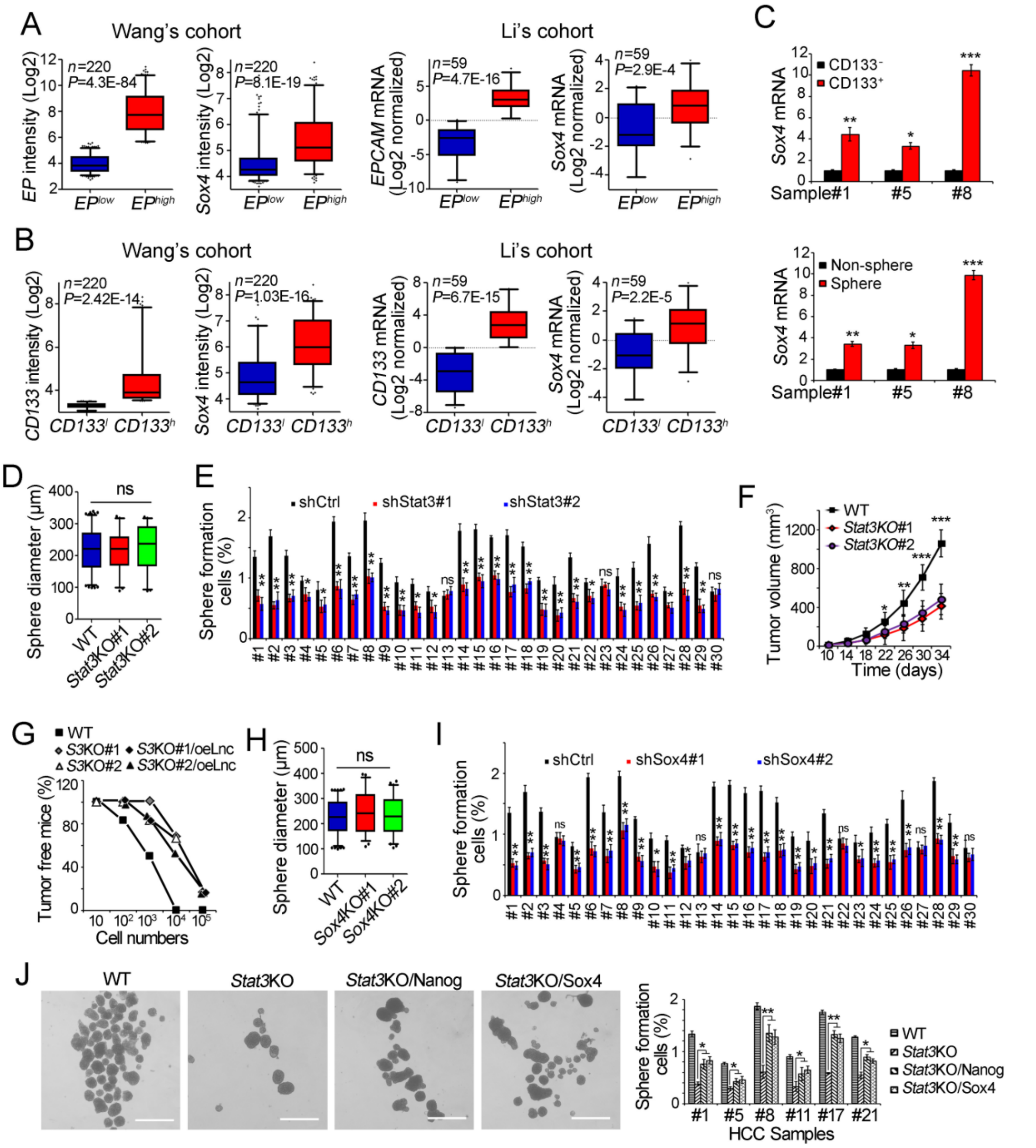

Supplementary Figure 5. Stat3/Sox4 signaling is required for liver TIC self-renewal. (A, B) Sox4 expression levels were correlated with EPCAM (A) and CD133 (B) expression according to Wang's cohort (left panel) and Li's cohort (right panel). R language and Bioconductor were used for gene expression levels. Then the samples were divided into two groups according to EPCAM (A) and CD133 (B) expression levels, and Sox4 expression levels were analyzed. The cut-off values were the mean values of EPCAM (A) and CD133 (B) expression levels. (C) Sox4 was highly expressed in liver TICs. Sox4 mRNA expression levels were determined using real-time PCR, showing high Sox4 expression in CD133+ liver TICs (upper panel) and oncospheres (lower panel). (D) Diameter of Stat3 deficient or WT spheres. Primary sample #1 cells were used for sphere formation. ns, not significant. Stat3KO, Stat3 knockout. (E) Sphere formation ratios of 30 primary samples. 30 primary HCC samples were infected

with Stat3-silenced or control pSiCoR lentivirus. 5000 Stat3-silenced or control cells were used for sphere formation. (F) Stat3 is required for tumor propagation. 1×10^6 indicated cells were subcutaneously injected into BALB/c nude mice and tumor volumes were measured every four days. (G) 10, 1×10^2, 1×10^3, 1×10^4 and 1×10^5 indicated cells were subcutaneously injected into BALB/c nude mice for tumor formation. The ratios of tumor-free mice were shown. (H) Diameter of Sox4 deficient or WT spheres. Primary sample #1 cells were used for sphere formation. ns, not significant. Sox4KO, Sox4 knockout. (I) Sphere formation ratios of *Sox4*-silenced or control primary cells. For Sox4 knockdown, 30 primary HCC samples were infected with Sox4-silenced or control pSiCoR lentivirus, followed by flow cytometer sorting. 5000 Sox4-silenced or control cells were used for sphere formation. (J) Stat3 deficient cells were established using CRISPR/Cas9 approach, followed by Nanog and Sox4 rescue. The established cells were incubated with sphere formation medium for 2 weeks. Typical images were shown in the left panel and calculated sphere formation ratios were shown in the right panel. Scale bars, 500μm. For A, B, D, H, data were shown as box and whisker plot. Box, interquartile range (IQR); whiskers, 5–95 percentiles; horizontal line within box, median. For C, E, F, I, J, data were shown as means ± SD. Two-tailed Student's t test was used for statistical analysis. *$P < 0.05$; **$P < 0.01$, ***$P<0.001$.

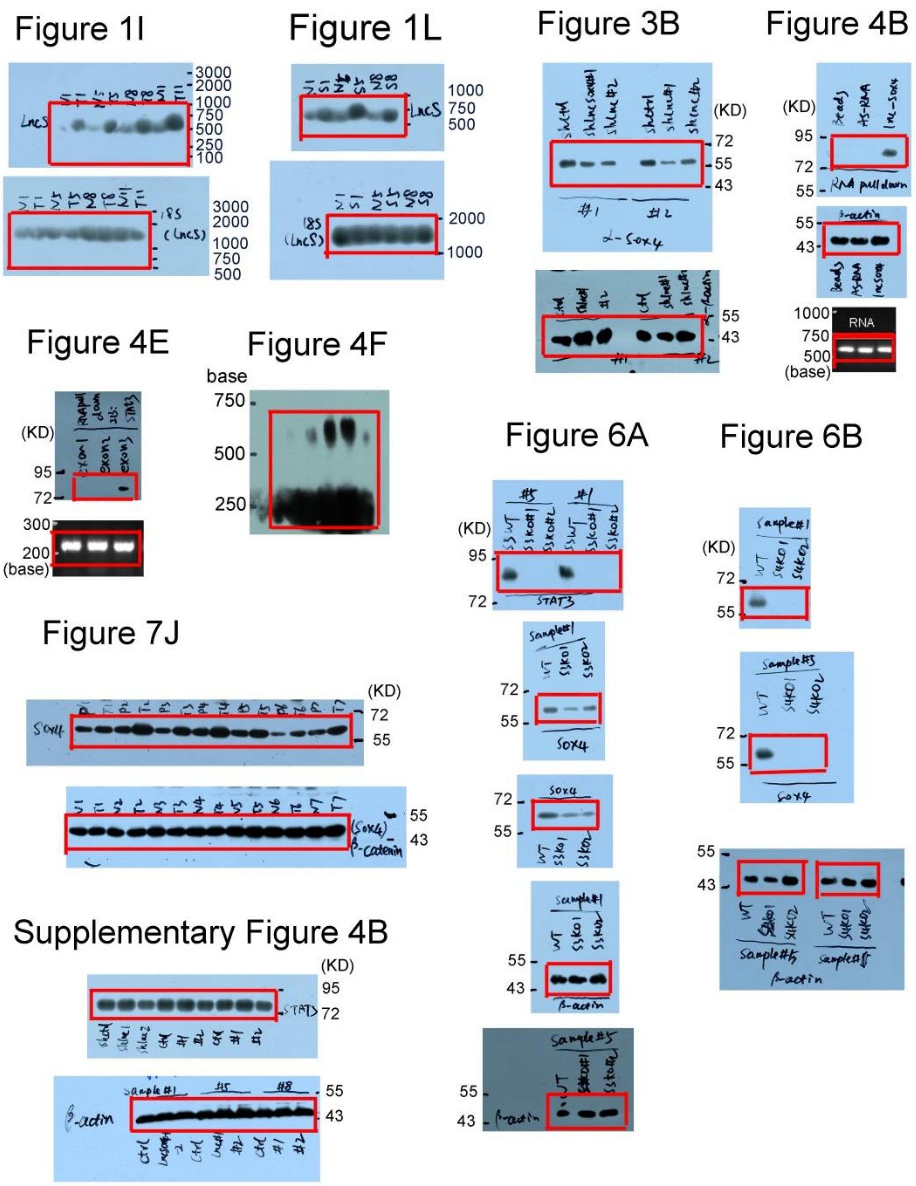

Supplementary Figure 6. Full blots and gels of figures. The red sections indicate blot results shown in the indicated figures.

Supplementary Table 1. TIC ratios of the indicated cells.

A

Cell	TIC ratio (95% CI)	*P* value
CD133+ (A)	1/384 (1/942-1/157)	
CD133- (B)	1/122867 (1/341055-1/44263)	7.2E-19 (B vs A)

B

Cell	TIC ratio (95% CI)	*P* value
WT (A)	1/8046-1/1264 (1/3091)	
sh*Lnc*#1 (B)	1/33426-1/4483 (1/12241)	0.02 (A vs B)
sh*Lnc*#2 (C)	1/80461-1/12651 (1/31905)	7E-5 (A vs C)

C

Cell	TIC ratio (95% CI)	*P* value
CD133+/shCtrl (A)	1/384 (1/942-1/157)	
CD133-/shCtrl (B)	1/122867 (1/341055-1/44263)	7.2E-19 (B vs A)
CD133+/shLnc (C)	1/8459 (1/21398-1/3344)	9.2E-7 (C vs A)
CD133-/shLnc (D)	1/184204 (1/593564-1/57165)	0.59 (D vs B)

D

Cell	TIC ratio (95% CI)	*P* value
shCtrl (A)	1/8046-1/1264 (1/3091)	0.74 (A vs C)
shLnc (B)	1/80461-1/12651 (1/31905)	4E-4 (B vs C)
shLnc/rcSox4 (C)	1/9424-1/1572 (1/3849)	

E

Cell	TIC ratio (95% CI)	*P* value
oeVec (A)	1/8046-1/1264 (1/3091)	
oeLnc (B)	1/1109-1/190 (1/459)	0.001 (A vs B)
oeVec/Sox4 KO(C)	1/94248-1/15727 (1/38499)	
oeLnc/Sox4 KO(D)	1/80461-1/12651 (1/31905)	0.74 (C vs D)

F

Cell	TIC ratio (95% CI)	*P* value
WT (A)	1/1223 (1/3340-1/448)	
Sox4KO#1 (B)	1/31905 (1/80461-1/12651)	5.7E-7 (B vs A)
Sox4KO#2 (C)	1/38499 (1/94248-1/15727)	1.7E-7 (C vs A)

G

Cell	TIC ratio (95% CI)	*P* value
CD133+/shCtrl (A)	1/384 (1/942-1/157)	
CD133+/shStat3 (B)	1/12241 (1/33426-1/4483)	7.4E-8 (B vs A)
CD133+/shSox4 (C)	1/26597 (1/69798-1/10135)	4.8E-12 (C vs A)
CD133-/shCtrl (D)	1/122867 (1/341055-1/44263)	7.2E-19 (D vs A)
CD133-/shStat3 (E)	1/167267 (1/517764-1/54037)	0.69 (E vs D)
CD133-/shSox4 (F)	1/184204 (1/593564-1/57165)	0.59 (F vs D)

10, 1×10^2, 1×10^3, 1×10^4 and 1×10^5 indicated cells were subcutaneously injected into BALB/c nude mice on the back and tumor formation was observed three months later. Liver TIC ratios was calculated using extreme limiting dilution analysis. CI, Confidence interval; vs, versus.

Supplementary Table 2. Information of clinical patients.

Sample No.	Age	Gender	Diagnosis	Tumor Size (cM)	Stage
1#	57	Female	HCC	2×2×2	Early
2#	43	Male	HCC	2.5×2.5×2.5	Early-Middle
3#	30	Male	HCC	5×5×4	Middle- Advanced
4#	70	Female	HCC	2×1.5×1.5	Early-Middle
5#	36	Male	HCC	7×5×2	Advanced
6#	62	Female	HCC	2×2×1.5	Early-Middle
7#	56	Male	HCC	1.2×1.2×1.2	Early-Middle
8#	71	Male	HCC	2×2×2	Advanced
9#	57	Female	HCC	2.5×2.5×2.5	Middle- Advanced
10#	33	Male	HCC	5×5×5	Early-Middle
11#	62	Male	HCC	8×8×8	Early
12#	72	Male	HCC	4×4×4	Early-Middle
13#	56	Female	HCC	3.5×3.5×3.5	Middle- Advanced
14#	55	Male	HCC	4×2×0.3	Advanced
15#	32	Male	HCC	12×2×2	Early-Middle
16#	70	Male	HCC	2.2×2.2×2.2	Middle- Advanced
17#	47	Female	HCC	5×4×3.5	Advanced
18#	44	Male	HCC	6×7×6	Early-Middle
19#	61	Male	HCC	6.5×4×3	Early-Middle
20#	65	Male	HCC	1.5×1.5×1.5	Advanced
21#	65	Male	HCC	10×8×7	Advanced
22#	52	Male	HCC	2.5×2×2	Middle- Advanced
23#	59	Male	HCC	5×3.5×3	Advanced
24#	62	Male	HCC	3×3×3	Middle- Advanced
25#	60	Male	HCC	5.5×5.5×5.5	Advanced
26#	62	Male	HCC	6×3×2	Advanced
27#	49	Female	HCC	5×5×2	Middle- Advanced
28#	52	Male	HCC	2×2×2	Middle- Advanced
29#	68	Male	HCC	7×6×6	Early-Middle
30#	58	Male	HCC	1.5×1.5×1.5	Advanced

Supplementary Table 3. Realtime PCR primers used in this study

Primers	Sequences
18S (Forward)	5'-AACCCGTTGAACCCCATT-3'
18S (Reverse)	5'-CCATCCAATCGGTAGTAGCG-3'
actin (Forward)	5'-TCCATCATGAAGTGTGACGT-3'
actin (Reverse)	5'-GAGCAATGATCTTGATCTTCAT-3'
LncSox4 (Forward)	5'-TTAGGGAAAGCCTTCTTTAGGGAT-3'
LncSox4 (Reverse)	5'-CTCCCAGCCCCTATTCCTTT-3'
AK000168 (Forward)	5'-GGAGCATCAATAGCTACTGG-3'
AK000168 (Reverse)	5'-CATTTCATTCCATTTTTCTG-3'
AA234127 (Forward)	5'-CAGGAGAAAGGTGTTGTTATGTC-3'
AA234127 (Reverse)	5'-AGATGCAGGTGATGCAGCGAC-3'
AW770635 (Forward)	5'-CAATTACACTCTACGAGATTAC-3'
AW770635 (Reverse)	5'-GTTAAAATACTGATTCTGAC-3'
AA092193 (Forward)	5'-GAAGGCAAAGTTCCTCTGCAG-3'
AA092193 (Reverse)	5'-CTCCTTTCTGTCTGGACCTC-3'
AW805892 (Forward)	5'-CAGCAGCTGGAAGGAAGATG-3'
AW805892 (Reverse)	5'-CTTCTCTCCCAGGGTACAAG-3'
AK128058 (Forward)	5'-GTGCTGAAGAGTTTGATGAC-3'
AK128058 (Reverse)	5'-TTGCCTGACGTAGAGAGTCCAG-3'
AL038637 (Forward)	5'-AGGTATTAAGCCCCACATGCAT-3'
AL038637 (Reverse)	5'-AAATTCTTGCCTTTGATACCTC-3'
AA584732 (Forward)	5'-CAACTGAGATGATGCCAGAG-3'
AA584732 (Reverse)	5'-CAAGCCTAGTTTTATCTTGAG-3'
BQ345206 (Forward)	5'-ACAGAGGGCCCATTTTTCTG-3'
BQ345206 (Reverse)	5'-CACAGTTCAGCAGCTGGTAT-3'
VEGF (Forward)	5'-ATCACGAAGTGGTGAAGTTC-3'
VEGF (Reverse)	5'-TGCTGTAGGAAGCTCATCTC-3'
Bcl2l1 (Forward)	5'-GAGCTGGTGGTTGACTTTCTC-3'
Bcl2l1 (Reverse)	5'-TCCATCTCCGATTCAGTCCCT-3'
Hif1a (Forward)	5'-GAACGTCGAAAAGAAAAGTCTCG-3'
Hif1a (Reverse)	5'-CCTTATCAAGATGCGAACTCACA-3'
Cox2 (Forward)	5'-ATGCTGACTATGGCTACAAAAGC-3'
Cox2 (Reverse)	5'-TCGGGCAATCATCAGGCAC-3'
Birc5 (Forward)	5'-CCAGATGACGACCCCATAGAG-3'
Birc5 (Reverse)	5'-TTGTTGGTTTCCTTTGCAATTTT-3'
MMP2 (Forward)	5'-CCCACTGCGGTTTTCTCGAAT-3'
MMP2 (Reverse)	5'-CAAAGGGGTATCCATCGCCAT-3'
c-Myc (Forward)	5'-GGCTCCTGGCAAAAGGTCA-3'
c-Myc (Reverse)	5'-CTGCGTAGTTGTGCTGATGT-3'
Twist1 (Forward)	5'-GTCCGCAGTCTTACGAGGAG-3'
Twist1 (Reverse)	5'-GCTTGAGGGTCTGAATCTTGCT-3'
Axin2 (Forward)	5'-TACACTCCTTATTGGGCGATCA-3'
Axin2 (Reverse)	5'-TTGGCTACTCGTAAAGTTTTGGT-3'
Ccnd2 (Forward)	5'-TTTGCCATGTACCCACCGTC-3'
Ccnd2 (Reverse)	5'-AGGGCATCACAAGTGAGCG-3'
Sox4 (Forward)	5'-AGCGACAAGATCCCTTTCATTC-3'
Sox4 (Reverse)	5'-CGTTGCCGGACTTCACCTT-3'
Nkd1 (Forward)	5'-GGGAAACTTCACTCCAAGCC-3'
Nkd1 (Reverse)	5'-CTCCCGATCCACTCCTCGAT-3'
Tcf7 (Forward)	5'-CTGGCTTCTACTCCCTGACCT-3'

Tcf7 (Reverse)	5’-ACCAGAACCTAGCATCAAGGA-3’
Hes6 (Forward)	5’-AGCAGGAGCCTGACTCAGTT-3’
Hes6 (Reverse)	5’-AGCTCCTGAACCATCTGCTC-3’
Hey1 (Forward)	5’-GTTCGGCTCTAGGTTCCATGT-3’
Hey1 (Reverse)	5’-CGTCGGCGCTTCTCAATTATTC-3’
Hes1 (Forward)	5’-TCAACACGACACCGGATAAAC-3’
Hes1 (Reverse)	5’-GCCGCGAGCTATCTTTCTTCA-3’
Nrarp (Forward)	5’-TCAACGTGAACTCGTTCGGG-3’
Nrarp (Reverse)	5’-ACTTCGCCTTGGTGATGAGAT-3’
Gli1 (Forward)	5’-TGGATATGATGGTTGGCAAGTG-3’
Gli1 (Reverse)	5’-ACAGACTCAGGCTCAGGCTTCT-3’
Ptch1 (Forward)	5’-CCACAGAAGCGCTCCTACA-3’
Ptch1 (Reverse)	5’-CTGTAATTTCGCCCCTTCC-3’
Gli3 (Forward)	5’-GAAGTGCTCCACTCGAACAGA-3’
Gli3 (Reverse)	5’-GTGGCTGCATAGTGATTGCG-3’
-4917 (Forward) (Sox4p)	5’-AAGCGAAAGGAGAGGTTCGG-3’
-4779 (Reverse) (Sox4p)	5’-GTTTCCTTCAGGCAGAACGC-3’
-4477 (Forward) (Sox4p)	5’-CCCTGTTACTCGTTGAGCGT-3’
-4348 (Reverse) (Sox4p)	5’-CGAGGTAGGAAATCGTGGGG-3’
-4099 (Forward) (Sox4p)	5’-ACTGTGTGCTAGTCACTGGT-3’
-3916 (Reverse) (Sox4p)	5’-GGGGTGCGTGCATTGAAAAA-3’
-3648 (Forward) (Sox4p)	5’-AGGTCTGACTAGCGCCTACA-3’
-3537 (Reverse) (Sox4p)	5’-AGGGAGGGACCTGAATGGAA-3’
-3202 (Forward) (Sox4p)	5’-CAGAGGGAACACTGCGTACT-3’
-3030 (Reverse) (Sox4p)	5’-TGCACACTGGAATGTGTTGGA-3’
-2665 (Forward) (Sox4p)	5’-GGCCAGTGGCTGTGTTTTAG-3’
-2580 (Reverse) (Sox4p)	5’-GGGAAGAGTGGAAGTTAAAGCAG-3’
-2150 (Forward) (Sox4p)	5’-ACAGATCCAGTCAGATGGCTAC-3’
-2080 (Reverse) (Sox4p)	5’-TAGGCCCAGGTTGTCAGACT-3’
-1834 (Forward) (Sox4p)	5’-GGCATCCTTTCTTTGCCACA-3’
-1753 (Reverse) (Sox4p)	5’-GCAACCCTGCCATTCCATTG-3’
-1182 (Forward) (Sox4p)	5’-GGCCACATCCCCATAGTTACA-3’
-1109 (Reverse) (Sox4p)	5’-ATGGGGCCTCCGGATTCATA-3’
-807 (Forward) (Sox4p)	5’-CCTGTTTGGGCTATGCAGGAT-3’
-695 (Reverse) (Sox4p)	5’-AAGGCAGGGAAGAGGACTTTG-3’
-490 (Forward) (Sox4p)	5’-TGCACCAGAGGCTGATTCTT-3’
-361 (Reverse) (Sox4p)	5’-TAACAAGGGGCTTGGAACGG-3’
-145 (Forward) (Sox4p)	5’-TCTCATTGCACGCGGAGATT-3’
-66 (Reverse) (Sox4p)	5’-TTCCTCATGCCAAACCCCTC-3’
-4700 (Forward) (Cox-2p)	5’-CCCTTCTTTCCTTCTCTGTAT-3’
-4500 (Reverse) (Cox-2p)	5’-ACAGAAGTCAACAATGTCTGTA-3’
-4300 (Forward) (Cox-2p)	5’-TTGTCTTTTAGTGTGACTTCTA-3’
-4100 (Reverse) (Cox-2p)	5’-AAGAGTGGAATGAAAAGCACTT-3’
-3900 (Forward) (Cox-2p)	5’-AATATTATAAAATAATTTCTCT-3’
-3700 (Reverse) (Cox-2p)	5’-ATTGATGAATTTCTGGAGGTTC-3’
-3500 (Forward) (Cox-2p)	5’-GAAGTGACAATTTCCAAGCTC-3’
-3300 (Reverse) (Cox-2p)	5’-GCCAAAGTAGTTGAATGAGTTG-3’
-3100 (Forward) (Cox-2p)	5’-CAAAACTTCTTGTGTAGCTAAG-3’
-2900 (Reverse) (Cox-2p)	5’-CCTTGTCTTCTTTCTTTCATGG-3’
-2700 (Forward) (Cox-2p)	5’-CACATGAATGGCTTATCACTTC-3’
-2500 (Reverse) (Cox-2p)	5’-TTTTACTGCCCCCTTCTGCTG-3’
-2300 (Forward) (Cox-2p)	5’-ACAATAGTCACAGTACTTTTC-3’
-2100 (Reverse) (Cox-2p)	5’-GGGCAGAGTTTTAGAAAGAAGT-3’

-1900 (Forward) (Cox-2p)	5'-TTCAGACAACAGAACAATTAAT-3'
-1700 (Reverse) (Cox-2p)	5'-TTCATTTTAAAGGAGTAGGTTG-3'
-1500 (Forward) (Cox-2p)	5'-AAAACAGTTAAAAAAAAAACCT-3'
-1300 (Reverse) (Cox-2p)	5'-TATATACATATATATATACACA-3'
-1100 (Forward) (Cox-2p)	5'-TGAAAAGTAAATGCTATGTTGT-3'
-900 (Reverse) (Cox-2p)	5'-GGACGCTAAATGTCCAAAACGT-3'
-700 (Forward) (Cox-2p)	5'-AAGCAACTTAGCTACAAAGATA-3'
-500 (Reverse) (Cox-2p)	5'-CACGTCGGGACAGACTGGGGCG-3'
-300 (Forward) (Cox-2p)	5'-AGAAAAGACATCTGGCGGAA-3'
-100 (Reverse) (Cox-2p)	5'-CCTCCTCTCCCCTTAAAAAAAT-3'
-4700 (Forward) (Birc5p)	5'-GCAGTGAGCCGAGATCATGC-3'
-4500 (Reverse) (Birc5p)	5'-AGGTGGAAGGATCACTTGATCC-3'
-4300 (Forward) (Birc5p)	5'-CCCGGATAATTTTTTGTATTT-3'
-4100 (Reverse) (Birc5p)	5'-GCCTGGGCAACACTGAGACC-3'
-3900 (Forward) (Birc5p)	5'-TCGGGAAGCACTGCAGATGGG-3'
-3700 (Reverse) (Birc5p)	5'-TTTCCTCCCACAATAGTTGGGT-3'
-3500 (Forward) (Birc5p)	5'-CACACCCGGCTATTTTTTTGT-3'
-3300 (Reverse) (Birc5p)	5'-GGCAACAAGAGTGAAACTCTG-3'
-3100 (Forward) (Birc5p)	5'-CCCACCTCAGCCTCCCAAAGT-3'
-2900 (Reverse) (Birc5p)	5'-AGTACATATCATTCCTGCGATC-3'
-2700 (Forward) (Birc5p)	5'-CAAGTTATGCGTCTAGACATG-3'
-2500 (Reverse) (Birc5p)	5'-TCCCTGAGAAGCAGAGTGAGGC-3'
-2300 (Forward) (Birc5p)	5'-CATTTGATGACATTGTGTGC-3'
-2100 (Reverse) (Birc5p)	5'-TACATGCGCCCGCCACCATGCC-3'
-1900 (Forward) (Birc5p)	5'-GGAGGTGGTGCGTCCTTGGT-3'
-1700 (Reverse) (Birc5p)	5'-CCCGAAATACTTCATTCTCTTA-3'
-1500 (Forward) (Birc5p)	5'-AGAGAGGGAAGGGGTAAGAGA-3'
-1300 (Reverse) (Birc5p)	5'-GCTGGGATTACAGATGTGAGCC-3'
-1100 (Forward) (Birc5p)	5'-AGATCATGCCACTGCACTCCA-3'
-900 (Reverse) (Birc5p)	5'-GCCCCACGCAGGCCTGGTGCCT-3'
-700 (Forward) (Birc5p)	5'-AGCACAGGCCCCCACCCTCCAC-3'
-500 (Reverse) (Birc5p)	5'-AGACGGGCATGAAGGACAAATG-3'
-300 (Forward) (Birc5p)	5'-CACCACGCCCAGCTAATTTTTG-3'
-100 (Reverse) (Birc5p)	5'-GTCCCTGCCCACACCTAGCGCC-3'
-4700 (Forward) (Ascl2p)	5'-CGGGCAGCACAGCTCCCTGTGG-3'
-4500 (Reverse) (Ascl2p)	5'-GAAGTGGGGGGCCTTACCCAG-3'
-4300 (Forward) (Ascl2p)	5'-AAGGAGGCCGAGTTCAACCTGC-3'
-4100 (Reverse) (Ascl2p)	5'-TTTAACCACTTAAGAATTTAAA-3'
-3900 (Forward) (Ascl2p)	5'-GATTGCGCCACTGCACTCCAGC-3'
-3700 (Reverse) (Ascl2p)	5'-TTTAACCTTTTGAGGGACCACC-3'
-3500 (Forward) (Ascl2p)	5'-TCACCCATAAAAAGAGAGGAAAT-3'
-3300 (Reverse) (Ascl2p)	5'-CTTCCCATGACCCTTCCCCCAG-3'
-3100 (Forward) (Ascl2p)	5'-GACCTGGACTTCATGTTTCAGT-3'
-2900 (Reverse) (Ascl2p)	5'-CTATCTCCGACTTCATTCCTGA-3'
-2700 (Forward) (Ascl2p)	5'-AAGAAACCACGTACCCTTTCAC-3'
-2500 (Reverse) (Ascl2p)	5'-GACGAAGCTGGTGAACCTCGAG-3'
-2300 (Forward) (Ascl2p)	5'-CCAGTCCAGGAGACAGCATGCT-3'
-2100 (Reverse) (Ascl2p)	5'-CTTCATCCTCTTCCCAATCTGG-3'
-1900 (Forward) (Ascl2p)	5'-AGGGGCTTGGACGAAGACCGGG-3'
-1700 (Reverse) (Ascl2p)	5'-CCCCTCGGTCATCTGTTGCTGT-3'
-1500 (Forward) (Ascl2p)	5'-GGAGCAGAGGCAAGCCCGCATC-3'
-1300 (Reverse) (Ascl2p)	5'-AGGGGCCGGAAGAGTAGCACCT-3'
-1100 (Forward) (Ascl2p)	5'-AGCTGGGTCCACAAAAATCTCC-3'

-900 (Reverse) (Ascl2p)	5'-GGTGGGGCCTGAGAGTGCAAAC-3'
-700 (Forward) (Ascl2p)	5'-GGAGGGCAGGACGGGCTGGAGG-3'
-500 (Reverse) (Ascl2p)	5'-GCTTTCCCCGTCCCTCCACGCG-3'
-300 (Forward) (Ascl2p)	5'-AGGGCGGGGCCCAGCAGGAACC-3'
-100 (Reverse) (Ascl2p)	5'-CGCGCAGCCCACACCCACGCCC-3'
-4700 (Forward) (Tcf7p)	5'-CTCTGACTCTAGACTCCTTCCT-3'
-4500 (Reverse) (Tcf7p)	5'-TGGCCAAATTGGCTTGAGAGGT-3'
-4300 (Forward) (Tcf7p)	5'-ACCTGATGCAGAGTAGCAGCAT-3'
-4100 (Reverse) (Tcf7p)	5'-CCTCTCAGGGCAAAACCCGGTT-3'
-3900 (Forward) (Tcf7p)	5'-TCAGAAAAGGTCAGAGTTCACG-3'
-3700 (Reverse) (Tcf7p)	5'-TAATGGGTGCTCTTGAACGAGA-3'
-3500 (Forward) (Tcf7p)	5'-ACTGCCCAAATCTAAAGCTGGA-3'
-3300 (Reverse) (Tcf7p)	5'-TCAGGACCAGGACCAGCTGCAA-3'
-3100 (Forward) (Tcf7p)	5'-AAGGTCTTGGGCCCCCTATACA-3'
-2900 (Reverse) (Tcf7p)	5'-AACTATCACACTCTAGTGTTCC-3'
-2700 (Forward) (Tcf7p)	5'-CACATAGTAATCACTCAGTATA-3'
-2500 (Reverse) (Tcf7p)	5'-GGGTGGCTTCCTGAAGGAGGTA-3'
-2300 (Forward) (Tcf7p)	5'-AGCAAGCCTTCAGACTGATTTC-3'
-2100 (Reverse) (Tcf7p)	5'-ACCCTCAAGTTTCTTTGGACAT-3'
-1900 (Forward) (Tcf7p)	5'-AAAAAGAACCTCACTGTTGTTG-3'
-1700 (Reverse) (Tcf7p)	5'-GAAAGGGAATCCGGATCTGCCC-3'
-1500 (Forward) (Tcf7p)	5'-GCAGGGTCCCCCTCATCTCCAT-3'
-1300 (Reverse) (Tcf7p)	5'-AAGAGCTAGGCCATGCTTCCCT-3'
-1100 (Forward) (Tcf7p)	5'-TGCTAGGGGAGCTGCTGTTGAC-3'
-900 (Reverse) (Tcf7p)	5'-TCTAACCCTCGCTCGATCCAGG-3'
-700 (Forward) (Tcf7p)	5'-GAGGTCCGGTGCTCTTGGCTG-3'
-500 (Reverse) (Tcf7p)	5'-CTTGCCCCATCCTCCATCCCTG-3'
-300 (Forward) (Tcf7p)	5'-GCCGGAGGAGAAACCTGGGCGC-3'
-100 (Reverse) (Tcf7p)	5'-AGAGGCCGGGACGCGCCCACTC-3'

Sox4p: *Sox4* promoter

Cox-2p: *Cox-2* promoter

Birc5p: *Birc5* promoter

Ascl2p: *Ascl2* promoter

Tcf7p: *Tcf7* promoter

Supplementary Table 4. shRNA sequences used in this study

shRNA	Sequence
shAK000168	5'-GGACTAACGTTTAGAGAAG-3'
shAA234127	5'-GGAAGACTGAGCCTCTATA-3'
shAW770635	5'-GACTTATCACAGGTCCTAA-3'
shAA092193	5'-GCCCAACCTGTAACTACAA-3'
shAW805892	5'-GCCAGATTGTATCATCACT-3'
shAK128058	5'-GGACTACGATAAGCTGAAG-3'
shAL038637	5'-GAGGTATCAAAGGCAAGAA-3'
shAA584732	5'-GAGCCAACTTCAATTATAA-3'
shBQ345206	5'-GCCTTCATGTATTTATCAA-3'
sh*LncSox4*#1	5'-GGATGACAAGAAGTACAAA-3'
sh*LncSox4*#2	5'-GGATGAAAAGCTAACTACA-3'
shStat3#1	5'-GCAGCAGCTGAACAACATG-3'
shStat3#2	5'-GCATCTGCCTAGATCGGCTA-3'
shSox4#1	5'-GCTGGAAGCTGCTCAAAGA-3'
shSox4#2	5'-GCGACAAGATCCCTTTCAT-3'
